# Essential laboratory tests for medical education

**DOI:** 10.1016/j.acpath.2022.100046

**Published:** 2022-09-13

**Authors:** Andrea T. Deyrup, Danielle D'Ambrosio, Jeannie Muir, Laboratory Test Working Group, Andrea Deyrup, Barbara Knollmann-Ritschel, Danielle D'Ambrosio, Jeannie Muir, Teresa Scordino, Matthew Kraswoski, Liyun Cao, Kinjal Shah, Jennifer Zepf, Samuel Grindstaff, Ashley Inman, Karen Moser, Kristin Olson, Lynette Parker, Aaron Shmookler, Joyce Ou, Angelica Putnam, Luisa Watts, Elham Vali Betts, Scott Lovitch, Kristen Stashek, Melina Flanagan, Nirupama Singh, Eric Suarez, Ellen Dudrey, Mary Furlong, Marta Margeta, Adam Wilberger, Joanna Chan, Amy Lin, Barbara Knollmann-Ritschel

**Affiliations:** a Department of Pathology, Duke University School of Medicine, Durham, NC, USA; b Department of Pathology, Uniformed Services University of the Health Sciences, Bethesda, MD, USA; c Department of Pathology, Walter Reed National Military Medical Center, Bethesda, MD, USA; d Laboratory Test Working Group, Undergraduate Medical Education Section of the Association of Pathology Chairs, Wilmington Delaware, USA

**Keywords:** Laboratory testing, Pathology, Medical education, Curriculum, Pathology competencies in medical education, Lab tests, Undergraduate medical education

## Abstract

Medical practice requires physicians to have a broad understanding of the basic sciences, have competent clinical skills, and have an ability to practice in evolving health systems in a cost-effective and evidence-based manner. Essential to the practice of medicine is an understanding of the common laboratory tests and the ability to use them effectively. The Essential Laboratory Tests for Medical Education (ELTME) is a concise document explaining the pathophysiology of common laboratory tests and clinical context for each test and was developed in response to an expressed need from medical students, residents and fellows, and medical educators. The ELTME is linked to the Pathology Competencies in Medical Education and its third competency of diagnostic medicine and therapeutic pathology. The ELTME table is a document of common laboratory tests in alphabetical listing. Laboratory tests may be easily queried by name or organ system, and with simple editing tools, new tables may be constructed to fit the needs of individual curricula or learners. Furthermore, the table may be easily expanded by educators who wish to add specific tests to complement their curricula.

## Background

Medical education requires the teaching of knowledge skills and attitudes essential for the competent practice of medicine. The field of medicine has changed rapidly over the last decades, with an exponential increase in the knowledge and skills necessary for effective practice in the modern environment. The skill of understanding laboratory testing is often overlooked, despite the fact that 70% of medical decisions rely on the accurate ordering and interpretation of laboratory tests.^1^

While laboratory testing is only a small sliver of the cost of healthcare in the United States, nearly 3/4 of health care costs are influenced by that same laboratory testing.^2^ Value-based care is a core component of the Health Systems Sciences^3^ curriculum and, therefore, laboratory testing must be viewed in the context of a systems model. Although health care delivery can be seen as one domain and knowledge as another, laboratory testing is integrally involved with both. In health care delivery, laboratory testing plays a role in the clinical domain including diagnosis, treatment, and prevention of diseases, among others. The knowledge domain includes pathophysiology and data collection.

In recognition of the importance of laboratory medicine, the 2017 Pathology Competencies in Medical Education (PCME),^4^ dedicated its third competency to diagnostic medicine and therapeutic pathology. The authors recognized that physicians must understand 1) which questions must be asked in the care of the patient; 2) which specific laboratory tests can assist in answering those questions; and 3) how to accurately interpret laboratory test results for optimal patient care and safety. The third PCME competency includes learning goals and objectives across clinical and anatomic pathology including general principles, transfusion medicine, hematology, microbiology, chemistry, immunology, genomics, and anatomic topics including autopsy, surgical pathology, and cytopathology.

Our goal with the Essential Laboratory Testing for Medical Education (ELTME) is to provide educators and learners with an easy-to-use summary of the pathophysiology and clinical context (“clinical pearls”) associated with common laboratory tests. We hope this publication will facilitate incorporation of laboratory testing in medical school curricula. We anticipate medical students, residents, and fellows will find this publication useful as a reference to better order and interpret laboratory tests and to provide superior patient care.

## Methods

A formal proposal to form a working group that would create a resource for undergraduate medical education was approved by the Association of Pathology Chairs Undergraduate Medical Education Section (UMEDS) Council. One goal of the project was to help medical students understand basic principles of laboratory testing to improve decision making and patient safety. Another goal was to create a useful resource that would allow medical educators to readily incorporate basic laboratory test information into their curricula. The project planned to link common laboratory tests to the third competency of the PCME, and to highlight pathophysiology and clinical pearls of those common laboratory tests.

A question regarding the teaching of laboratory values was posed on the UMEDS listserv July 7, 2020. As the question generated much discussion on this topic, a formal working group was presented to and approved by the UMEDS Council on August 24, 2020. A template for pathophysiology and clinical pearls was created with examples, and the initial laboratory list included approximately 250 laboratory tests generated from a survey of the Mayo Clinic Laboratory testing website.^5^ Follow up emails were sent to UMEDS members who had expressed interest in participating in the project in October 2020. Additional tests were added to the list based on the recommendations from UMEDS members. Once working group members had submitted their contributions for each laboratory test, clinical pathology editors and clinical colleagues were identified to review the submissions and recommend changes. Laboratory tests were correlated with the PCME, and revisions condensed the current laboratory test list to 181 common laboratory tests. The table was edited to create a uniform product with similar style and content depth for all entries. Table 1 includes the laboratory tests of the ELTME.

**Table 1:** Essential laboratory tests.

Test	PCME	Reference values	Pathophysiology	Clinical Pearls	References 5, 6, 7, 8, 9, 10 are used when not otherwise specified.	Organ system/Panel
25-Hydroxyvitamin D2 and D3, serum	CHEM 1.4	20–50 ng/mL (optimal)	When UVB strikes the epidermis, 7-dehydrocholesterol is converted into previtamin D3, which is next converted into vitamin D3 (cholecalciferol). In the liver, vitamin D3 is hydroxylated to form 25-hydroxyvitamin-D3. In the kidney, it is finally converted into the biologically active form: 1,25-dihydroxyvitamin-D3. Initial laboratory assessment for vitamin D status analyzes levels of 25-hydroxyvitamin-D3, not the biologically active form; testing for 1,25-dihydroxyvitamin D may be indicated in the setting of renal disease. Vitamin D promotes calcium absorption in the gut and helps maintain normal serum calcium and phosphate. Vitamin D can be obtained by sunlight, diet, or supplements.	In children, vitamin D deficiency is associated with rickets, osteomalacia, muscle pain/weakness and tetany (due to hypocalcemia). In adults with vitamin D deficiency, risk of osteoporosis, osteomalacia and fracture is increased. Vitamin D deficiency is common, particularly with decreasing sun exposure. Clinical interpretation of vitamin D nutritional status typically uses total 25-hydroxyvitamin D levels, although some analytical methods can separately determine 25-hydroxyvitamin D2 and D3.		Skeletal
ABO type, blood	TM1.1	A, B, AB, O	The ABO system is essential in transfusion medicine and encompasses the four common blood types: A, B, AB, and O. The A and B codominant alleles encode homologous enzymes forming their respective defining terminal sugar after the first few months of life. Absence of A and B antigens leads to O blood type, the precursor antigen for which is the H system.	The majority of the world's population are of blood type O. “universal donor” O red blood cells (RBCs) lacking A and B antigens are used in emergent transfusions when the blood type is unknown. Antibodies to A antigen (anti-A) and/or B antigen (anti-B) are naturally occurring and cause severe acute hemolytic transfusion reactions: Anti-A will lyse type A or AB cells, while anti-B will lyse type B or AB cells. Subgroups of B and especially A antigen are evaluated prior to solid organ transplantation.		Transfusion
Acetaminophen, serum	CHEM 1.7	10-25 μg/mL^a^	Acetaminophen (N-acetyl-p-aminophenol or APAP) is used to relieve pain and reduce fever. It is processed in the liver, predominantly to sulfate and glucuronide. A smaller portion is metabolized by several cytochrome P-450 isoforms (most notably CYP2E1), which convert APAP to the reactive, toxic metabolite N-acetyl-p-benzoquinone imine (NAPQI). Normally, NAPQI is detoxified through glutathione conjugation and subsequently excreted in urine. When supratherapeutic doses are ingested, glutathione becomes depleted and excess NAPQI forms protein adducts, which initiate oxidative stress pathways, ultimately resulting in mitochondrial dysfunction and hepatocellular damage.	Acetaminophen is rapidly absorbed from the gastrointestinal tract; the utility of serum measurements is dependent on the pharmacokinetics of immediate versus extended-release formulations. Peak serum concentrations are detectable as early as 1–2 h after ingestion (immediate release) or greater than 4 h after ingestion (extended release). Management of acetaminophen overdoses includes oral or IV N-acetylcysteine (NAC), which functions as a glutathione substitute and binds directly to NAPQI. Acetaminophen is a component of many medications which contributes to the risk of accidental overdose. In the United States, acetaminophen toxicity accounts for about 50% of cases of acute liver failure. Serum acetaminophen concentrations and estimated time of ingestion are used in decisions on antidote treatment.	^11^	Toxicology
Acetylcholine (ACh) receptor antibodies, serum	IMM 1.4	≤0.02 nmol/L (binding antibodies) Negative (modulating antibodies)	The ACh receptor (AChR) is found on the surface of muscle cells at the neuromuscular junction. AChR autoantibodies are found in ˜85% of patients with generalized and ˜50% with ocular myasthenia gravis (MG). MG is an autoimmune disorder characterized by skeletal muscle weakness and increased fatigability. Autoantibodies to AChR are classified as binding, blocking, or modulating. Binding antibodies cause complement activation that destroys the AChR. Blocking antibodies prevent the binding of ACh. Modulating antibodies crosslink the receptor subunits, resulting in internalization.	AChR binding antibody titer does not necessarily correlate with disease severity, but changes in titer can be useful in monitoring response to treatment in individual patients. A negative result does not rule out the diagnosis of MG: 30 to 40% of patients with MG who are negative for AChR antibodies express a muscle-specific kinase (MuSK) autoantibody. False positives may occur in the setting of thymoma (without MG), Lambert-Eaton myasthenic syndrome, small cell lung carcinoma and penicillamine treatment.	^12^	Nervous
Activated partial thromboplastin time (aPTT), plasma	H 2.1	25–37 s	aPTT assesses the coagulation factors of the intrinsic (factors XII, XI, IX, and VIII) and the common (factors X, V, II, and fibrinogen) pathway. The test is performed by measuring time to clot formation when a surface activator, phospholipids, and calcium are added to the patient's platelet-poor plasma.	Deficiency of any of the assessed factors can cause elevations of aPTT. When both PT and aPTT are elevated, the deficiency is in the common pathway. A prolonged aPTT should be interpreted in the context of a concurrent PT to determine whether the deficiency is in the intrinsic pathway or in the common pathway. A mixing study may be performed to distinguish a factor deficiency from inhibition. Heparin and antiphospholipid antibodies (lupus anticoagulant) cause isolated aPTT elevation. For most aPTT reagents, the factor VIII activity must be below 35–45% before changes in aPTT are noted. Since aPTT is a clot-based assay, anticoagulation therapy can result in elevated aPTT.		Coagulation
Activated protein C (aPC) resistance, plasma	H 2.1	Ratio ≥2.3	Protein C is a vitamin K-dependent clotting factor synthesized in the liver that is activated by thrombin. Activated PC inhibits coagulation by cleaving factors Va and VIIIa and by inactivating plasminogen activator inhibitor, leading to fibrinolysis. aPC also cleaves FV, which increases its anticoagulant activity. Resistance to aPC is most commonly seen in the context of the factor V Leiden (FVL) mutation in which an amino acid substitution removes the aPC cleavage site in both FV and FVa. The consequence is a prothrombotic state since the coagulant activity of FVa is not impeded and the anticoagulant activity of FV is not enhanced.	The FVL mutation increases the risk of venous thromboembolism in both heterozygotes and homozygotes. This assay reports the ratio of the aPTT with and without additional of exogenous aPC and is highly sensitive and specific. Diagnosis can also be made by genetic testing which may be indicated in suspected familial thrombophilia or when the patient is anticoagulated.		Coagulation
ADAMTS13 activity, plasma	H 2.1	≥70%	ADAMTS13 is a circulating metalloproteinase protease synthesized primarily by the liver. It cleaves ultra-large multimers of von Willebrand factor (vWF), thereby disrupting platelet aggregation. An IgG autoantibody to ADAMTS13 inhibits enzyme activity, preventing vWF cleavage and resulting in a prothrombotic state in thrombotic thrombocytopenic purpura (TTP). This assay reports ADAMTS13 activity as a percentage of the activity seen in wildtype individuals.	TTP is characterized by thrombocytopenia, microangiopathic hemolytic anemia (intravascular hemolysis with schistocyte formation), fever, renal dysfunction and neurological deficits. It can be inherited or, more commonly, acquired. In the latter, ADAMTS13 activity is typically <10%. Decreased ADAMTS13 activity can also be seen in liver disease, sepsis, disseminated intravascular coagulation and pregnancy.		Coagulation
Adrenocorticotropic hormone (ACTH), plasma	CHEM 1.3	7.2–63 pg/mL a.m. draws	Corticotropin releasing hormone (CRH) from the hypothalamus induces ACTH synthesis in the adenohypophysis. The main function of ACTH is to stimulate cortisol secretion by the adrenal gland. CRH release is circadian resulting in an ACTH peak between 6 and 8 a.m. and a trough around 11 p.m. CRH may also be released in response to stress and hypoglycemia. There is tight feedback control between the adrenal glands, pituitary gland, and hypothalamus to regulate the secretion of ACTH, arginine vasopressin and CRH. Measuring levels based on ACTH's diurnal secretory pattern may aid in determining ACTH-dependent Cushing disease.	Elevated cortisol levels can be seen in Cushing disease (pituitary ACTH-secreting tumor), Cushing syndrome, ectopic ACTH-secreting tumor and adrenal hyperplasia. A dexamethasone suppression test can help distinguish Cushing disease from other causes of hypercortisolism. Important causes of hypocortisolism include primary and secondary adrenal insufficiency and congenital adrenal hyperplasia.		Endocrine
Alanine aminotransferase (ALT; aka serum glutamate pyruvate transaminase), serum	CHEM 1.4	Males: 7–55 U/L Females: 7–45 U/L	ALT is an enzyme normally present in the cytoplasm of hepatocytes. With plasma membrane injury, it is released and enters the blood. Low levels of ALT may be released with damage to the kidney and skeletal and cardiac muscle. ALT is more specific for the liver than aspartate aminotransferase (AST).	Both ALT and AST levels increase in liver disease; however, ALT is more specific for liver injury and remains elevated longer than AST. Increases in ALT may precede symptom onset. In inflammatory conditions of the liver (e.g., acute viral hepatitis, autoimmune hepatitis), ALT levels are usually equal to or higher than the increase seen in AST, resulting in an ALT:AST ratio of more than 1. In the setting of excess alcohol use, AST is elevated to a greater extent than ALT, leading to an AST:ALT ratio of >2. In end-stage liver disease, both enzymes may be low due to massive tissue destruction.		Hepatic/Comprehensive Medical Panel (CMP)
Albumin, serum	CHEM 1.4	3.5–5 g/dL	Albumin is the predominant serum protein and is made in the liver. It is the main protein for maintaining whole blood oncotic pressure and can be decreased in liver disease, malabsorption, burns or due to loss in nephrotic syndrome.	A low albumin may be indicative of poor prognosis in renal disease as it reflects a loss of renal filtering capability.		CMP
Aldosterone, serum	CHEM 1.3	Adults: ≤21 ng/dL	Aldosterone is the main mineralocorticoid produced by the adrenal cortex. Aldosterone production and secretion are controlled through the renin-angiotensin-aldosterone system. Aldosterone stimulates sodium transport in the distal renal tubules and is a key regulator of blood pressure and blood volume.	Conditions that increase aldosterone include adrenal adenoma, adrenal hyperplasia and excessive activation of the renin-angiotensin-aldosterone system (e.g., renin-producing tumor, renal artery stenosis). Aldosterone deficiency can be seen with low renin (such as in renal disease) or with high renin (from primary adrenal insufficiency).		Endocrine
Alkaline phosphatase (AP), serum	CHEM 1.4	Varies with age and sex. Adult males: 40–129 U/L Adult females: 35–104 U/L	Alkaline phosphatase is an enzyme from the cellular membrane. The major isoenzymes are liver, bone, and placental. The liver isoenzyme remains constant throughout life, the bone isoenzyme (from osteoblasts) is predominant in the growing years, and the placental isoenzyme rises during pregnancy, then falls off quickly after parturition. In non-pregnant adults, the liver isoenzyme is the predominant isoenzyme.	Alkaline phosphatase is a sensitive marker for biliary disease or metastasis to the liver. The intestinal isoenzyme is usually a smaller fraction of the total, but in patients with blood types O or B, the level may be elevated after meals and account for up to 25% of the alkaline phosphatase level. The most common causes of elevated alkaline phosphatase are related to the liver (e.g., biliary obstruction, metastases, primary sclerosing cholangitis) and bone (e.g., Paget disease, hyperparathyroidism, metastases). Other causes of increased alkaline phosphatase include malignancies, chronic inflammatory conditions (e.g., sarcoidosis, ulcerative colitis) and sepsis.		Hepatic, Skeletal, CMP
Alpha 1 antitrypsin (AAT), serum	GE 2.1	100–190 mg/dL	The alpha-1 antitrypsin (AAT) protein is produced by hepatocytes and inhibits neutrophil serine proteases, most notably neutrophil elastase. AAT deficiency is caused by AAT mutations that result in protein misfolding and accumulation in the liver, resulting in hepatocyte damage. Consequent low AAT levels in lung alveolar cells renders them vulnerable to destructive proteases, increasing risk for emphysema (panacinar), especially at an early age.	Inherited forms (autosomal recessive, co-dominant) of AAT deficiency are thought to be underdiagnosed, with the most common clinically relevant form (PiZZ type, with loss of up to 90% serum AAT) estimated to affect 1 in 2500 to 1 in 5000 individuals in the US and Europe. AAT serum measurements and protease inhibitor (Pi) phenotyping are important parts of the diagnostic work-up for symptomatic patients. In addition, both gene-targeted testing (single and multigene panel) as well as comprehensive genomic testing are available for genotyping of S and Z alleles and rare pathogenic variants. Liver dysfunction can be seen in patients with certain allele combinations (e.g., PiMZ), and rare patients present with necrotizing panniculitis.	^13^^,^^14^	Hepatic
Alpha-fetoprotein (AFP), serum	CHEM 1.4, 1.8	<8.4 ng/mL	AFP is a glycoprotein synthesized during development by embryonic hepatocytes and fetal yolk sac cells. In the fetus, its function is analogous to that of albumin and it is the most abundant serum protein. Production drops after birth; however, it is produced by some tumors, especially germ cell tumors.	AFP is elevated in acute liver injury and hepatocellular carcinoma (HCC). In acute liver injury modest increases (100–200 ng/dL) reflect regenerating hepatocytes. Serum AFP levels are increased in 70% of patients with HCC and can be used to monitor therapeutic response and recurrences; however, the test lacks sensitivity and specificity for diagnosis of early disease. AFP is also increased in germ cell tumors of the ovary and testis (e.g., yolk sac tumor, embryonal carcinoma), but is negative in pure seminoma and choriocarcinoma. AFP is elevated in maternal serum in the setting of open neural tube defects (e.g., anencephaly, spina bifida). Pregnant people are typically screened at 15–20 weeks gestation.		Tumor marker, Neuro
Amylase, serum	CHEM 1.4	28–100 U/L	Amylase hydrolyzes complex carbohydrates and is primarily secreted by the salivary glands and the pancreas. Amylase is increased with gland inflammation or duct obstruction.	Amylase is increased in acute pancreatitis, pancreatic pseudocyst, pancreatic duct obstruction (e.g., choledocholithiasis, pancreatic cancer). In acute pancreatitis, amylase levels increase rapidly (3–6 h of symptom onset) and remain elevated for about 5 days; lipase is currently the preferred test for the diagnosis of acute pancreatitis due to higher specificity. Amylase levels are decreased in pancreatic insufficiency and chronic pancreatitis. Other rarer sources of increased amylase include anorexia (due to salivary gland hyperplasia), small bowel injury, salpingitis or hepatitis among others.		Pancreatic
Androstenedione, serum	CHEM 1.3	Varies with age, sex and sexual development Adult males: 40–150 ng/dL Adult females: 30–200 ng/dL	Androstenedione is a steroid hormone produced from cholesterol in the testes, adrenal cortex, and ovaries. Androstenedione production in the adrenal glands is controlled by the adrenocorticotropic hormone (ATCH), whereas in the gonad it is controlled by luteinizing hormone and follicle-stimulating hormone. Androstenedione is a precursor of testosterone.	Androstenedione is increased in hirsutism, polycystic ovary syndrome (PCOS), virilizing adrenal tumors, precocious puberty, Cushing disease, ectopic ACTH-producing tumors and congenital adrenal hyperplasia. Androstenedione can be decreased in hyperlipidemia, psychosis, psoriasis, hypopituitarism, glucocorticoid treatment, and with increasing age.		Endocrine
Anti-centromere antibody, serum	IMM 1.2	<1.0 U	Anti-centromere antibodies arise when nuclear fragments, typically from apoptotic cells, are not effectively cleared, thereby eliciting autoantibodies. These antigens are not typically accessible to the immune system and, therefore, are recognized as non-self. Anti-centromere antibodies are associated with a speckled antinuclear antibody (ANA) pattern.	Anti-centromere antibodies are present in approximately 80% of cases of limited cutaneous scleroderma/CREST syndrome (calcinosis, Reynaud phenomenon, esophageal dysmotility, sclerodactyly, and telangiectasia). These antibodies are not specific and can also be present in systemic sclerosis and systemic lupus erythematosus.		Autoimmunity
Anti-citrullinated peptide antibody, serum	IMM 1.2	<20 U	Citrullinated proteins represent post-translational modifications that can be associated with inflammation, particularly in synovial tissues. In rheumatoid arthritis (RA), autoantibodies are induced against a number of citrullinated antigens. These anti-citrullinated peptide antibodies (ACPA) have been identified in synovial fluid of some RA patients, and may play a pathogenic role by triggering proinflammatory cytokines and bone destruction via osteoclastogenesis.	ACPA can be found in 60–80% of patients with RA. ELISA-based serum tests show specificity ranging from 85 to 99%, which is higher than rheumatoid factor. ACPA may precede RA symptoms by several years. ACPA levels may correlate with disease progression and response to anti–tumor necrosis factor-alpha antibody treatment.	^15^	Autoimmunity, Skeletal
Anti-DNA topoisomerase I (Scl-70) antibody, serum	IMM 1.2	<1.0 U	DNA topoisomerase I is present in the nucleolus and nucleoplasm and its function is to cleave and relax supercoiled DNA. Anti-DNA topoisomerase I antibodies arise when nuclear fragments, typically from apoptotic cells, are not effectively cleared, thereby eliciting autoantibodies. Anti-DNA topoisomerase antibodies produce a fine-speckled nuclear or nucleolar ANA pattern.	Anti-DNA topoisomerase antibodies are found in 20–60% of patients with systemic sclerosis/scleroderma. They are associated with the diffuse variant of the disease, pulmonary fibrosis and poor prognosis.		Autoimmunity
Anti-double-stranded (ds) DNA antibody, serum	IMM 1.2	<30 IU/mL	Anti-ds DNA antibodies arise when nuclear fragments, typically from apoptotic cells, are not effectively cleared, thereby eliciting autoantibodies. These antibodies form immune complexes that fix complement and cause damage to kidneys, skin, and other tissues. Anti-ds DNA antibodies produce a peripheral or diffuse staining pattern.	Anti-ds DNA antibodies are seen in systemic lupus erythematosus (fairly sensitive and specific for SLE) and in Sjögrens yndrome or mixed connective tissue disease. Anti-dsDNA IgG levels appear to correlate with disease activity and renal involvement in SLE. Anti-ds DNA antibodies are rare in other rheumatologic diseases but may be seen.		Autoimmunity
Anti-histone antibody, serum	IMM 1.2	<100 AU/mL^a^	Anti-histone antibodies arise when nuclear fragments, typically from apoptotic cells, are not effectively cleared, thereby eliciting autoantibodies. Histones are the nuclear proteins that bind to DNA and help condense it into chromatin. Anti-histone antibodies produce a homogenous (diffuse) nuclear staining pattern.	Anti-histone antibodies are positive in drug-induced lupus, but they are not specific. Patients with low levels of the acetyltransferase that acetylates and detoxifies some drugs (e.g., hydralazine, procainamide) appear to be more likely to develop a positive antinuclear antibody after exposure to these drugs.		Autoimmunity
Anti-mitochondrial antibody (AMA), serum	IMM 1.2	<0.1 U	AMAs are seen in 95% of patients with primary biliary cholangitis (PBC) with high specificity (98%). An infectious etiology with molecular mimicry to mitochondrial antigens has been hypothesized. The most common autoantigen is the E2 subunit of pyruvate dehydrogenase (PDC-E2). The role of AMAs in the pathogenesis of PBC is unclear; disease severity and rate of progression do not correlate with antibody titer. B and T cells autoreactive for PDC-E2 are seen in PBC.	On indirect immunofluorescence, AMAs have a coarse, granular cytoplasmic pattern. AMAs are both sensitive and specific for PBC, though they are rarely positive in CREST (calcinosis, Raynaud phenomenon, esophageal hypomotility, sclerodactyly, and telangiectasia) syndrome.		Autoimmunity
Anti-neutrophil cytoplasmic antibody (ANCA) – myeloperoxidase (MPO), serum	IMM 1.2	Negative	ANCAs are predominantly IgG that bind Fcγ receptors in neutrophils. MPO is found in the granules of neutrophils and the lysosomes of monocytes; MPO-ANCA activates both cell types. MPO-ANCA was formerly known as pANCA (perinuclear-ANCA) based on indirect immunofluorescent staining. Positive indirect immunofluorescence results should be followed by ELISA testing for the specific antibody target.	The ANCA-associated vasculitides are a group of small-vessel necrotizing vasculitis syndromes that include granulomatosis with polyangiitis (GPA); microscopic polyangiitis (MPA); and eosinophilic granulomatosis with polyangiitis (EGPA). EGPA is typically associated with MPO-ANCA. Both MPO-ANCA and PR3-ANCA may be seen in MPA, though MPO-ANCA is more common.		Autoimmunity, Vascular
Anti-neutrophil cytoplasmic antibody (ANCA) – proteinase 3 (PR3), serum	IMM 1.2	Negative	ANCA are predominantly IgG that bind Fcγ receptors in neutrophils. PR3-ANCA was formerly known as cANCA (cytoplasmic-ANCA) based on indirect immunofluorescent staining. The primary target of these antibodies is proteinase 3 (PR3) in the cytoplasm of neutrophils. Positive indirect immunofluorescence results should be followed by ELISA testing for the specific antibody target.	The ANCA-associated vasculitides are a group of small vessels necrotizing vasculitis syndromes that include granulomatosis with polyangiitis (GPA); microscopic polyangiitis (MPA); and eosinophilic granulomatosis with polyangiitis (EGPA). PR3-ANCA is positive in the majority of cases of GPA.		Autoimmunity, Vascular
Anti-nuclear antibody (ANA), serum	IMM 1.2	≤1.0 U	ANAs arise when nuclear fragments, typically from apoptotic cells, are not effectively cleared, thereby eliciting autoantibodies. This test detects serum levels of antibodies against multiple nuclear and cytoplasmic cellular structures. ELISA is more specific but is less sensitive. ANAs are present in many autoimmune diseases but are not specific; they can be seen in other conditions (e.g., infection, malignancy) or in otherwise healthy individuals. Different autoantibodies produce different ANA patterns on immunofluorescence (e.g., homogeneous, speckled, centromere, nucleolar).	Further testing to refine the diagnosis is based on the pattern of ANA staining and the patient's clinical symptoms. The clinical setting should guide selection of further autoantibody testing. The American College of Rheumatology generally recommends against testing for ANA subserologies if ANA is negative.		Autoimmunity
Anti-nucleosome antibody (anti-chromatin), serum	IMM 1.2	<1.0 Negative AI	Antinucleosome antibodies arise when nuclear fragments, typically from apoptotic cells, are not effectively cleared, thereby eliciting autoantibodies. These antigens are not typically accessible to the immune system and, therefore, are recognized as non-self. Nucleosomes are subunits of the histone-DNA complex.	Antinucleosome antibodies are positive in up to 75% of patients with systemic lupus erythematosus (SLE) and up to 100% of drug-induced lupus patients; in the former, they are associated with renal disease. Antibody titer correlates with disease severity.		Autoimmunity
Anti-phospholipid antibody (aPL; also known as lupus anticoagulant antibody), serum	IMM 1.4	Negative	aPLs are acquired, heterogeneous autoantibodies that bind negatively charged phospholipid proteins. They are frequently seen in patients with systemic lupus erythematosus. aPLs prolong the aPTT by binding phospholipids, a necessary cofactor in this test. In vivo, however, aPLs may cause arterial and venous thrombosis and are associated with antiphospholipid syndrome (multiple thrombi and multiple late spontaneous abortions).	aPL testing is complex and incorporated in the workup of patients with suspected antiphospholipid syndrome. aPTT (>PT) is typically prolonged in the presence of aPL due to prolonged time to form a clot in vitro due to binding of aPL to the antiphospholipid surface. Although the in vitro test suggests anticoagulation, aPL are prothrombotic in vivo and the term lupus anticoagulant is misleading.	^16^	Autoimmunity, Coagulation
Anti-Ro (anti-SS-A) antibody, serum	IMM 1.2	<1.0 U	These antibodies are a type of anti-nuclear autoantibody against cellular proteins. Depending on the technique used for detection, these antibodies are present in 40–80% of patients with primary Sjögren syndrome. They can be found in 50% of patients with SLE as well as subacute cutaneous lupus erythematosus (SCLE), neonatal lupus and primary biliary cholangitis.	In recent years, there has been increasing literature regarding the significance of serum anti-52-kD Ro and anti-60-kD Ro; the former has a reported association with congenital heart block and the latter is more often present in autoimmune disease.	17, 18, 19	Autoimmunity
Anti-Smith (Sm) antigen antibody, serum	IMM 1.2	<1.0 U	Sm antigens are part of a group of nuclear proteins called extractable nuclear antigens (ENAs). Other ENAs include SSA, SSB, and ribonuclear protein. Anti-Sm antibodies arise when nuclear fragments, typically from apoptotic cells, are not effectively cleared, thereby eliciting autoantibodies. Anti-Sm antibodies are associated with a speckled ANA pattern.	Anti-Sm antibodies are fairly specific for lupus; they are positive in 20–30% of SLE patients and tend to remain constant as the disease progresses.		Autoimmunity
Anti-smooth muscle antibody (ASMA), serum	IMM 1.2	Negative	ASMAs are associated with autoimmune hepatitis (AIH), though their role in pathogenesis is unknown. They are typically directed against filamentous actin (F-actin) in AIH.	ASMA are positive in approximately 50% of patients with type I autoimmune hepatitis. ASMA is more specific than ANA for AIH, particularly when titers are greater than 1:80. ASMA may be the only marker present in AIH. ASMA (and ANA) levels may fluctuate during treatment and may disappear with corticosteroid therapy. Antibody titer does not predict outcome.		Autoimmunity, Hepatic
Antibody screen, blood	TM1.1	Negative	Antibodies can be made against self-red blood cell (RBC) antigens (autoantibodies) and against foreign RBC antigens (alloantibodies) after exposure (e.g., blood transfusion, pregnancy, transplantation). In an antibody screen, a patient serum sample is mixed with reagent RBCs with known antigen profiles. When the antibody screen is positive, the blood bank identifies these antibodies. This process may also be performed for platelets.	The most clinically significant natural alloantibodies target A and/or B antigens, depending on the recipient's blood type. An ABO-incompatible RBC transfusion can result in an acute hemolytic transfusion reaction. A screen is valid for no more than 3 days.	^20^	Transfusion
Antithrombin (AT) activity, plasma	H 2.1	Adults: 80–130%	AT is an anticoagulant produced by hepatocytes that inactivates factors IIa, IXa and Xa. It also inhibits other serine proteases in the coagulation cascade to a lesser degree.	AT deficiency is a risk factor for venous thromboembolism and may be inherited or acquired (e.g., nephrotic syndrome, fulminant hepatic failure, disseminated intravascular coagulation). Since heparin acts as an anticoagulant by potentiating the activity of AT, AT deficiency leads to heparin resistance. When inherited thrombophilia is under consideration, a panel of tests is usually performed and typically includes AT activity, factor V Leiden mutation, prothrombin G20210A mutation, protein C activity/resistance, protein S activity, and free protein S antigen.		Coagulation
Arsenic, blood/hair	CHEM 1.7	Blood: <13 ng/mL Hair: <1.0 μg/g of hair	Arsenic is rapidly cleared from the circulation into the phosphate pool; therefore, blood levels are only useful for acute toxicity. However, arsenic has a high affinity for the sulfhydryl groups of the amino acid cysteine, which is highly prevalent in keratin-rich tissues such as hair and nails. Testing of hair samples or toenail clippings can document exposure and correlates with time of exposure.	Acute arsenic toxicity presents as arrhythmias and non-specific gastrointestinal symptoms such as diarrhea, nausea and vomiting. Chronic exposure typically manifests as hyperkeratosis, peripheral neuropathies, renal failure, anemia, liver dysfunction or cardiac arrhythmias. Chronic exposure is associated with an increased risk of cancers of the urinary bladder, liver, skin and lung. Hair from the nape of the neck is used to assess recent exposure; pubic or axillary hair is evaluated for long-term exposure.	^21^	Toxicology
Aspartate aminotransferase (AST; aka serum glutamate oxaloacetate transaminase), serum	CHEM 1.4	Males: 8–48 U/L Females: 8–43 U/L	AST is an enzyme normally present in the cytoplasm and mitochondria of hepatocytes. With hepatocyte membrane injury, it is released and enters the blood. In addition to the liver, AST can also be released from the kidney, skeletal muscle, and the heart. Alanine aminotransferase (ALT) is more specific for the liver than AST.	AST is increased in liver disease, toxic injury, or viral infections of the liver. In the setting of excess alcohol use, AST is elevated to a greater extent than ALT, leading to an AST:ALT ratio of >2. Both ALT and AST require vitamin B6 for activity, but ALT is more dependent on vitamin B6, which is often deficient in individuals who chronically drink excessively.		Hepatic, CMP
Basophil count, blood	H 4.2	0.01–0.08 × 10^9^/L	A basophil count is part of a complete blood count (CBC) with a differential. Basophils are the least common white blood cells (WBC) in the peripheral blood. They arise from myeloid precursor cells in the bone marrow. Basophils release histamine, cytokines, and other compounds to assist with immune responses.	Basophils are increased in myeloproliferative neoplasms (especially chronic myeloid leukemia), hypothyroidism, chronic inflammation and autoimmune diseases. Basopenia can occur in hyperthyroidism, infection and severe allergies.		CBC
Beta-2-microglobulin (B2M), serum	CHEM 1.8	1.21–2.70 μg/mL	B2M is the constant light chain of HLA Class I expressed on the surfaces of most nucleated cells. It is filtered in the glomerulus.	B2M is elevated in diseases such as B-cell non-Hodgkin lymphomas and plasma cell neoplasms. B2M levels are used for staging and monitoring treatment in myeloma though serum free light chain levels are more effective for the latter. Elevated B2M is a poor prognostic indicator in this disease. Serum B2M may also be elevated in patients on long-term hemodialysis, in whom B2M may deposit as amyloid. Risk of this complication has been decreased, but not eliminated, by improved hemodialysis protocols.	^22^	Immunologic, Tumor Marker
Bicarbonate, serum	CHEM 1.6	Males ≥18 years; Females ≥10 years: 22–29 mEq/L	Bicarbonate (HCO_3_^−^) is an important buffer in the body to prevent acid-base disorders and to maintain the proper pH of epithelial secretions. Luminal concentration is regulated by the cystic fibrosis transmembrane conductance regulator (CFTR). Bicarbonate is an important component of gastrointestinal mucus secretions, such as in the stomach, where it helps prevent gastritis and gastric ulcers. Renal bicarbonate excretion increases in alkalemia.	Serum bicarbonate concentration is used to calculate pH in the Henderson-Hasselbalch equation when evaluating an acid-base disorder. Bicarbonate levels are low in metabolic acidosis and respiratory alkalosis and elevated in metabolic alkalosis and respiratory acidosis. If serum levels are sufficiently high, metabolic alkalosis will result. Causes of increased serum bicarbonate include gastric HCl loss (e.g., vomiting) and K^+^ loss. *CFTR* mutations seen in cystic fibrosis lead to decreased bicarbonate levels in luminal secretions.	^23^	BMP/Chem 7
Bilirubin (total, direct and indirect),serum	CHEM 1.4	Total bilirubin, varies with age Adults: ≤ 1.2 mg/dL Direct bilirubin: 0.0–0.3 mg/dL	Bilirubin is the principal pigment in bile. 80% is derived from breakdown of aged red blood cells (RBCs); the remaining 20% is derived from destruction of heme-containing proteins (e.g., myoglobin, cytochromes, catalase) and from catabolism of heme. Total bilirubin is the sum of direct (conjugated) bilirubin, which is water soluble and excreted in urine, and indirect (unconjugated) bilirubin, which is not water soluble. There is a minor, generally clinically insignificant discrepancy when the total and direct bilirubin values are subtracted to obtain a value for indirect bilirubin.	Neonates are at risk for kernicterus (unconjugated hyperbilirubinemia) due to immature uridine diphosphoglucuronate glucuronosyltransferase (UGT), as well as increased erythrocyte destruction. Breast-fed infants are also at higher risk of kernicterus. Elevated total bilirubin in older children and adults can result from increased RBC destruction or decreased liver function.	^24^	Hepatic
Blood urea nitrogen (BUN), serum	CHEM 1.5	Males ≥18 years: 8–24 mg/dL Females ≥18 years: 6–21 mg/dL	Ammonia is generated when proteins are catabolized. The liver metabolizes this ammonia to urea, which is released into the blood and then eliminated by the kidneys, thereby removing nitrogen from the body.	BUN may be elevated due to liver damage or conditions affecting the urinary tract (e.g., acute glomerulonephritis, polycystic kidney disease, urinary obstruction due to benign prostatic hyperplasia [BPH]). Dehydration and congestive heart failure can lead to increased BUN, the latter due to decreased renal perfusion. BUN alone is not tremendously informative; these data are usually compared with creatinine to give a BUN/creatinine ratio which is more useful in monitoring renal health.		BMP/Chem 7, Hepatic, Renal
Brain natriuretic peptide or B-type natriuretic peptide (BNP), plasma N-terminal (NT)-pro hormone BNP (NT-proBNP), serum	CHEM 1.2	Varies with sex and age	In response to ventricular wall stretch and volume overload, cardiac myocytes cleave an N-terminal section from the BNP prohormone (NT-proBNP) to release active BNP and the inactive N-terminal fragment, NT-proBNP. BNP downregulates the renin-angiotensin-aldosterone system, decreases sympathetic tone in the heart and kidney, and increases renal blood flow and sodium excretion. NT-proBNP has a longer half-life than BNP.	NT-proBNP and BNP levels are useful in distinguishing acute onset dyspnea secondary to congestive heart failure (CHF) versus lung disease since it is elevated in the former, but not the latter. However, NT-proBNP should not be used in isolation to establish the diagnosis of CHF.		Cardiac
C-reactive protein (CRP), serum	IMM 1.1	≤8 mg/L	CRP is an acute phase reactant that acts as an opsonin. In acute inflammation, IL-6 stimulates production of CRP from hepatocytes. CRP is a sensitive but non-specific marker of inflammation. It has a short half-life (hours).	CRP is increased in a variety of acute illnesses and inflammatory conditions (e.g., bacterial infection, myocardial infarction). Higher baseline levels of plasma CRP are associated with increased risk of chronic heart disease and stroke, possibly through the inflammatory response associated with atherosclerosis. Assays to measure the low baseline levels of CRP are often referred to as ‘high-sensitivity’ or ‘cardiac’ CRP assays to distinguish from regular CRP assays that measure the much higher levels seen inflammation or infection.		Inflammatory
C3 complement, serum	IMM 1.1	75–175 mg/dL	Complement proteins are plasma proteins that are activated directly by microbes or by antibodies bound to antigens, and mediate important functions (opsonization, inflammation, lysis of some cells). Most are made by the liver and are decreased in severe liver disease or when widespread complement activation consumes these proteins. They are acute phase reactants and are increased in multiple disease states. The 3 complement activation pathways converge with the activation of the C3 protein. The C3b fragment coats the surface of the target cell, attracting phagocytes and continuing the complement cascade to cell lysis.	C3 complement is measured by immunoassay, which does not address its function, and is evaluated in the workup of autoimmune diseases (e.g., SLE). Levels often correlate with disease activity. Low levels of both C3 and C4 indicate classical pathway activation while low serum level of C3 with normal C4 level indicates alternative pathway activation. The C3a fragment is an anaphylatoxin that stimulates mast cell and basophil degranulation leading to increased vascular permeability and vasodilation. C3 deficiency leads to increased risk of pyogenic infections.	^25^	Inflammatory
C4 complement, serum	IMM 1.1	14–40 mg/dL	Complement proteins are plasma proteins that are activated directly by microbes or by antibodies bound to antigens, and mediate important functions (opsonization, inflammation, lysis of some cells). Most are made by the liver and are decreased in severe liver disease or when widespread complement activation consumes these proteins. They are acute phase reactants and are increased in multiple disease states. There are 3 complement activation pathways; C4 is part of the classical pathway and levels are low when this pathway is activated.	C4 complement is measured by immunoassay, which does not address its function. C4 evaluation is part of the workup of autoimmune diseases (e.g., SLE). Levels may help determine disease activity. The C4a fragment is an anaphylatoxin that stimulates mast cell and basophil degranulation leading to increased vascular permeability and vasodilation. C4 deficiency is associated with an increased risk of developing autoimmune diseases.	^25^	Inflammatory
Cadmium, urine/blood	CHEM 1.3	Urine: <3 mg/g creatinine Blood: <4.9 μg/L	Cadmium forms adducts with proteins, followed by protein denaturation and cell cycle disruption through indirect oxidative damage.	Chronic exposure to cadmium can be nephrotoxic, accumulating in the medulla and the proximal tubule and leading to tubulointerstitial nephritis and proteinuria. Common sources of cadmium exposure include tobacco smoke and prolonged spray painting without respiratory protection (e.g., auto body repair).	^26^	Toxicology
Calcitonin, serum	CHEM 1.3	Adult males ≤14.3 pg/mL Adult females ≤7.6 pg/mL	Calcitonin is secreted by parafollicular cells (C cells) of the thyroid gland in response to elevated ionized calcium concentration. Calcitonin inhibits the action of parathyroid hormone and also inhibits bone resorption by directly binding to osteoclasts. Calcitonin lowers serum calcium and phosphorus levels.	Calcitonin can be elevated in patients with medullary thyroid carcinoma, which may be seen in the setting of multiple endocrine neoplasia (MEN) syndrome type 2.		Endocrine
Calcium, serum	CHEM 1.3	Ionized (free) calcium: Adults: 4.57–5.43 mg/dL Total calcium: Adults: 8.6–10.0 mg/dL	Calcium binds to negatively charged sites on proteins and is affected by pH. Alkalosis leads to an increase in negative charge and binding and thus a decrease in free calcium. Acidosis leads to a decrease in negative charge and binding and thus an increase in free calcium. Decreased ionized calcium levels stimulate the parathyroid glands to secrete parathyroid hormone (PTH), which leads renal tubular cells to increase calcium absorption and drives osteoclasts to release calcium from bone. It also induces renal conversion of calcidiol to calcitriol, which increases intestinal absorption of calcium.	Serum calcium is 50% ionized (“free” or “active”), 40% bound to albumin, and 10% bound to other ions. “Total” calcium is a sum of these 3 components. Total calcium measures are affected by serum albumin levels; direct measurement of ionized calcium is more precise. Hypercalcemia can be seen in primary hyperparathyroidism (increased PTH secretion) or in malignancy (secretion of PTH-related proteins or by bone destruction from metastases). Other causes of hypercalcemia include drugs/supplements, endocrine disorders, granulomatous diseases. Common causes of hypocalcemia include chronic renal failure and hypomagnesemia due to impaired PTH secretion and PTH end-organ resistance. Hypoalbuminemia is the most common cause of pseudohypocalcemia.	^27^	Endocrine, Metabolic
Cancer antigen 125 (CA-125), serum	CHEM 1.8	<46 U/mL	CA-125 is a glycoprotein that is normally expressed on cells derived from coelomic epithelium (e.g., fallopian tube, ovary, colon).	Serum CA-125 is increased in advanced epithelial ovarian cancer and can be used to assess the presence of residual disease following debulking surgery or to monitor for recurrence. It should not be used as a screening test for ovarian cancer. CA-125 can also be elevated with pregnancy, non-malignant pathology such as endometriosis and pelvic inflammatory disease, and other non-gynecologic cancers.		Tumor marker
Cancer antigen 19-9 (CA19-9), serum	CHEM 1.8	<35 U/mL	CA 19-9 is an FDA-approved serum tumor marker for pancreatic ductal adenocarcinoma. It is the sialylated form of the Lewis (a) blood group antigen found on red blood cells (RBCs). It is shed from neoplastic pancreatic ductal cells into blood in ∼70–90% of patients with pancreatic ductal adenocarcinoma. CA 19-9 can be elevated in other malignancies (e.g., cholangiocarcinoma, colon cancer, gastric cancer, ovarian cancer).	In patients who are Lewis negative, tumor cells cannot produce CA 19-9, due to lack of the enzyme fucosyltransferase. CA 19-9 is not useful as a screening test as it can be elevated in benign liver disease and patients with cholestasis. In the setting of malignancy, high levels correlate with unresectable disease and a worse prognosis. It can be used to assess tumor response to treatment and presence of recurrence/metastasis if pre-treatment CA 19-9 levels are known.		Tumor marker
Carcinoembryonic antigen (CEA), serum	CHEM 1.8	Nonsmokers: ≤ 3.0 ng/mL Smokers: <5.0 ng/mL	CEA is an oncofetal antigen normally expressed during fetal development, but not in the tissue of healthy children or adults. It is expressed by certain epithelial malignancies (e.g., colorectal, pancreatic, and lung cancer).	Serum CEA elevations of >20 ng/mL are generally associated with malignancy; however, this should not be used as a screening test due to low sensitivity and specificity. It is useful for monitoring recurrence of colon cancer after resection.		Tumor marker
CD4 count, blood	M 3.4	Adults: 400–1400 CD4^+^ T cells/μL^a^ Pediatric ranges vary by age	CD4^+^ T-cell counts are considered the best indicator of the current state of immune competence in patients with HIV. CD4 counts may be directly measured by flow cytometry, or a percentage of CD4^+^ T-cells can be measured by flow cytometry and the absolute count calculated (CD4^+^ T-cell % x WBC count).	CD4 counts are not used for the initial diagnosis of HIV infection. They are used in patients with known HIV infection for disease staging, to assess risk for certain HIV-associated complications and the need for prophylaxis, and to evaluate response to antiretroviral therapy. Key clinical indicators include CD4 counts <200 cells/μL (*Pneumocystisjirovecii* prophylaxis indicated) and <50/μL (*Mycobacteriumavium* complex prophylaxis indicated). CD4 counts are typically performed at diagnosis and subsequently every 3–6 months; more frequently if counts are trending down. CD4 counts may also be performed as part of evaluation of other immune deficiency syndromes, typically as part of a more extensive panel to evaluate T-cell and/or B-cell populations.	^28^	Hematologic
Ceruloplasmin, serum	CHEM 1.4	Varies with age and sex Adult males: 19.0–31.0 mg/dL Adult females: 20.0–51.0 mg/dL	Ceruloplasmin is an acute-phase reactant synthesized by the liver and is the primary copper-carrying protein in the blood. It carries 95% of total copper in human plasma. In Wilson disease, a mutation in the *ATP7B* gene results in decreased copper transport into bile, decreased incorporation into ceruloplasmin and decreased ceruloplasmin secretion into blood. Copper accumulates in hepatocytes, which can cause injury by 1) the Fenton reaction; 2) binding to protein sulfhydryl groups; and 3) displacement of other metals from hepatic metalloenzymes.	Low ceruloplasmin levels can be seen in the setting of Wilson disease, Menkes disease, zinc toxicity, or malnutrition (trace mineral deficiency). Pathologic effects of excess copper include cirrhosis, neuropsychiatric symptoms, hematuria/proteinuria and Kayser-Fleischer rings.		Hepatic
Chloride, serum	CHEM 1.5	≥18 years: 98–107 mmol/L	Chloride is part of the basic metabolic panel (BMP: Cl^−^, Na^+^, glucose, BUN, K^+^, CO_2_, creatinine) and reflects the body's ability to maintain fluid homeostasis and acid-base balance. It is the primary anion in the extracellular fluid and is necessary for transmitting action potentials in neurons. Dietary salt intake is the main source of chloride for humans. Serum levels are partially regulated by the kidney, and reabsorption occurs by both passive and active processes.	When chloride levels are the only abnormality in the BMP, a metabolic disorder is most likely. Alkalosis is seen with low chloride levels while acidosis is associated with high chloride levels. Causes of decreased chloride include vomiting, diarrhea, diabetic ketoacidosis, syndrome of inappropriate antidiuretic hormone secretion (SIADH), metabolic alkalosis and heart failure. Low levels in patients with heart failure indicate advanced disease and decreased left ventricular ejection fraction. Causes of increased chloride include renal failure, dehydration, diabetes insipidus and respiratory alkalosis.	^23^^,^^29^	BMP/Chem 7
Cold agglutinin titer, serum	IMM 1.4	<1:64	Cold agglutinin syndrome is caused by IgM antibodies that bind red blood cells (RBCs) in peripheral parts of the body in which the temperature is < 37 °C. They may produce hemolysis or blood stasis, leading to cyanosis of the ears, fingers, and toes.	Blood specimen must be kept at 37-38 °C before testing. Autoimmune hemolytic anemia (AIHA) is caused by warm, cold or mixed-reactive antibody types. Cold antibody AIHA includes cold agglutinin disease (CAD) and paroxysmal cold hemoglobinuria (PCH). When cold antibody AIHA is suspected, a Coombs test for C3d antibodies should be performed; if this is positive, cold agglutinin titer and thermal activity should be performed. Titer does not correspond to disease activity. High titers can be seen in other conditions such as mycoplasma pneumonia, infectious mononucleosis and hematologic malignancies.	^30^	Coagulation
Complete blood count (CBC), blood	H 4.2	See individual tests	The CBC includes red blood cell (RBC), white blood cell (WBC) and platelet counts as well as all the RBC indices (mean corpuscular volume [MCV], mean corpuscular hemoglobin [MCH], mean corpuscular hemoglobin concentration [MCHC], RBC distribution width [RDW]). A CBC with differential includes all the above tests, plus a white blood cell differential count.	The CBC is a general screening test to evaluate overall health, to assess a wide range of hematologic disorders and to determine eligibility for medications and/or chemotherapy. See individual tests for further information.		CBC
Copeptin proAVP (arginine vasopressin), plasma	CHEM 1.3	Non-water deprived, non-fasting adults: <13.1 pmol/L Water deprived, fasting adults: <15.2 pmol/L	Neurosecretory cells in the hypothalamus secrete a pre-prohormone that is composed of AVP (also known as antidiuretic hormone, ADH), copeptin and neurophysin II; these three components are transported to the posterior pituitary. In response to decreased intravascular volume and increased plasma osmolarity/sodium concentration, AVP stimulates water reabsorption in the distal renal tubules.	When there is inadequate ADH, most commonly due to damage to the hypothalamus or pituitary stalk, diabetes insipidus results with consequent polyuria, polydipsia and hypernatremia. Syndrome of inappropriate antidiuretic hormone (SIADH) is when there is an inappropriate release of ADH leading to hyponatremia. This can occur in CNS disorders, lung disease or from a paraneoplastic syndrome caused by ectopic ADH secretion (e.g., small cell carcinoma of the lung). AVP has a short half-life in plasma, which makes analysis challenging; however, copeptin, which is secreted in equimolar amounts as AVP, has a longer half-life and, therefore, is used as a surrogate marker for AVP.	^23^^,^^31^	Endocrine
Copper, serum	CHEM 1.7	≥11 years old: 0.75–1.45 mg/mL	Copper is a component of multiple metalloproteins. About 90% of serum copper is complexed to ceruloplasmin with a smaller amount bound to transcuprein, metallothionein or albumin. Serum copper can be measured with mass spectrometry as free (non-ceruloplasmin-bound) serum copper or total (free and ceruloplasmin-bound) copper.	Low serum copper may be due to excess dietary iron or zinc or, less commonly, dietary deficiency (often in the setting of remote GI surgery such as gastric bypass) or Wilson disease. In Wilson disease, serum copper and ceruloplasmin may be normal due to hepatic injury, which releases these intracellular components into the blood. Free serum copper is a better means of diagnosing Wilson disease than is total serum copper due to variability in ceruloplasmin levels (e.g., pregnancy, inflammation).		Hepatic
Cortisol (free), serum	CHEM 1.3	5-25 μg/dL (morning collection) 2-14 μg/dL (afternoon collection)	Cortisol is the major endogenous glucocorticoid and is a key regulator of the stress response and glucose metabolism. Cortisol levels are regulated by adrenocorticotropic hormone (ACTH) from the pituitary gland in response to cyclic release of corticotropin releasing hormone (CRH) from the hypothalamus. Levels of ACTH and cortisol peak in the morning and trough in the late evening. In the serum, the majority of cortisol is bound to corticosteroid-binding globulin (CBG) and albumin.	Conditions that increase cortisol are known as hypercortisolism (Cushing syndrome); common causes include adrenal adenoma, pituitary adenoma (Cushing disease), ectopic ACTH production (e.g., from a tumor) and exogenous steroids. Symptoms associated with hypercortisolism include obesity, glucose intolerance and high blood pressure. Hypocortisolism (Addison disease) can be due to damage/disease of the adrenal glands or pituitary glands. Use of glucocorticoid medications will cause adrenal atrophy with reduction in endogenous cortisol production until medications are withdrawn.		Endocrine
Creatine kinase (CK; AKA creatine phosphokinase/CPK), serum	CHEM 1.2	Male: 39–308 U/L Female: 26–192 U/L	CK is the enzyme that phosphorylates creatine, generating phosphocreatine, an energy source abundant in the metabolically demanding tissues such as the brain, myocardium and skeletal muscle. There are several different CK isotypes with specialized subcellular and tissue distributions. The low CK level normally measurable in the blood reflects baseline leakage from skeletal muscle; abnormally elevated levels are due to the enzyme release from necrotic/injured myocardium or skeletal muscle.	CK elevation is one of the oldest markers of acute myocardial infarction, but it lacks specificity; currently, the more specific troponin test is used instead. Elevated CK remains a clinically useful marker of skeletal myonecrosis (e.g., due to muscular dystrophies, drug-induced/toxic myopathies, and autoimmune myopathies). In addition, CK levels can be elevated following infections, strenuous exercise, and crush injuries. Rhabdomyolysis (acute severe myonecrosis) is accompanied by markedly elevated CK levels (generally above 2000 U/L, although there is no absolute cutoff value).		Musculoskeletal
Creatinine, serum	CHEM 1.5	Males ≥15 years: 0.7–1.3 mg/dL Females ≥18 years: 0.6–1.0 mg/dL	Creatinine is derived from creatine (primarily synthesized in kidney and liver) and phosphocreatine (a quickly accessible energy source for brain and muscle). A relatively constant proportion of creatinine (related to skeletal muscle mass and metabolism) is released into blood, and creatinine is freely filtered by the glomerulus (with a minor contribution from renal tubular secretion). Serum creatinine has a nonlinear, inversely proportional relationship to glomerular filtration rate (GFR).	In addition to its role in GFR estimation, serum creatinine values can be used to evaluate kidney function in pathological states such as acute kidney injury. Both serum BUN and creatinine levels vary in inverse proportion to GFR, both increasing as GFR falls with the normal ratio being 10–15:1. When there is a disproportionate rise in BUN (higher ratio), it suggests pre-renal failure (e.g., dehydration, congestive heart failure). When the relative ratio is tilted toward creatinine, it is indicative of renal failure caused by intrinsic diseases of the kidney that affect GFR. Loss of muscle mass can lead to falsely low serum creatinine levels. Angiotensin-converting enzyme (ACE) inhibitors and angiotensin II receptor blockers (ARBs) can elevate serum creatinine.	^24^^,^^32^	Renal, BMP/Chem 7
Crossmatch, blood	TM1.1	Not applicable	If antibodies are identified in the antibody screen, donor red blood cells (RBCs) that are negative for the corresponding antigens reduce the risk of hemolysis. This test assesses donor red blood cell compatibility with recipient plasma by mixing patient serum with a sample of donor RBCs and is the final check prior to issuing a unit of RBCs from the blood bank for a patient. The process may also be performed for platelets.	Crossmatch reduces the general blood bank inventory by allocating specific units of blood (or platelets) to an intended recipient of a transfusion. This order is recommended for patients expecting a blood transfusion imminently or in some surgical cases. In an emergency, uncrossmatched RBCs may be used.	^20^	Transfusion
Cryoglobulins, serum	IMM 1.4	Negative	Cryoglobulins are immunoglobulins that precipitate at temperatures below 37°. There are three subtypes: Type I, monoclonal IgG or IgM; type II, a mixture of polyclonal and monoclonal immunoglobulins; and type III, polyclonal.	Type I cryoglobulins are associated with lymphoplasmacytic lymphoma and multiple myeloma. Type II cryoglobulins are seen in the setting of chronic hepatitis C and autoimmune disorders such as SLE. Type III cryoglobulins are seen in some autoimmune diseases and infections. At low temperatures, cryoglobulins can precipitate in the extremities or skin and occlude blood vessels, leading to purpura, skin necrosis, Raynaud phenomenon, arthralgias and neuropathy.		Coagulation
D-dimer, plasma	H 2.4	≤500 ng/mL fibrinogen equivalent units (FEU)	Thrombin cleaves fibrinogen to yield a fibrin monomer with a D domain on each end. The monomers polymerize to form a fibrin clot. D-dimers are a proteolytic byproduct of plasmin degradation of this clot and indicate 1) a fibrin clot was formed and 2) the clot was crosslinked by FXIIIa, followed by plasmin-mediated cleavage of the insoluble crosslinked fibrin clot.	Elevated D-dimer levels are seen in disorders marked by procoagulant and fibrinolytic activity (e.g., disseminated intravascular coagulation [DIC], deep venous thrombosis [DVT], pulmonary embolism [PE], recent surgery and trauma) and hypercoagulable states (e.g., pregnancy, liver disease, inflammation). A negative D-dimer result helps exclude DVT and PE; however a positive D-dimer is not specific. The extent of clotting/fibrinolysis does not correlate with the amount of D-dimers.		Coagulation
Deamidated gliadin IgG and IgA antibody, serum	IMM 1.4	Negative: <20.0 U Weak positive: 20.0–30.0 U Positive: > 30.0 U	Deamidated gliadin antibody is a serum test for celiac disease in patients with IgA deficiency (approximately 2% of patients with celiac disease) who therefore lack IgA antibodies against tissue transglutaminase (TTG), which is the first line serologic test for celiac disease. Celiac disease is an immune-mediated enteropathy of the small intestine triggered by gluten exposure in genetically susceptible individuals. Gliadin peptides are deamidated by tissue transglutaminase (tTG) and the deamidated fragments are presented to CD4^+^ T-cells, leading to activation/expansion of B-cells that secrete the deamidated gliadin antibody.	This test is used in IgA-deficient patients in whom anti-TTG (IgA) testing is not informative. While patients with celiac disease and IgA deficiency also make an IgG anti-tTG, IgG antibodies to deamidated gliadin are more sensitive/specific. The sensitivity of this test is reduced if patients are on a gluten-free diet prior to testing.	^33^	Gastrointestinal
Dehydroepiandrosterone sulfate (DHEAS), serum	CHEM 1.3	Varies with age	DHEA is the major adrenal androgen and is a precursor for sex steroids; the majority is secreted as a conjugate to sulfate, DHEAS. DHEA and DHEAS test results can be used interchangeably in most clinical situations.	DHEAS is secreted by the adrenal glands and is therefore a good marker for adrenal androgen production. Elevated DHEAS levels can cause symptoms or signs of hyperandrogenism in women though men are typically asymptomatic. DHEA/DHEAS are usually assessed in investigations of adrenal androgen production, such as the assessment of (1) hyperplasia, (2) adrenal tumors, (3) delayed puberty, and (4) hirsutism.		Endocrine
Desmoglein 1/3 antibody, serum	IMM 1.4	<20 RU/mL	Desmoglein 1 (DSG1) and desmoglein 3 (DSG3) are adhesion molecules on cell surface desmosomes. DSG1 is distributed from the stratum corneum to the basement membrane whereas DSG3 is limited to the lower portion of the epidermis. Autoantibodies to these proteins are seen in pemphigus foliaceous (DSG1) and pemphigus vulgaris (DSG3 and/or DSG1); binding activates complement, leading to disruption of intercellular adhesionsand blistering.	Pemphigus foliaceous is characterized by superficial blistering and rarely affects mucosal membranes. Pemphigus vulgaris is more common and shows more extensive blistering; the mucosa is frequently involved.		Skin
Direct antiglobulin test (DAT, direct Coombs test) Indirect antiglobulin test (indirect Coombs test), blood	TM 1.2	Negative	The DAT assesses in vivo coating of red blood cells (RBCs) by IgG and complement C3d, which can cause hemolysis resulting in anemia. IgG-bound RBCs are usually removed by the spleen, while complement-bound RBCs are destroyed by the membrane-attack complex. The direct Coombs test uses antihuman globulin to detect antibodies/complement that are bound to a patient's RBCs; the indirect Coombs test uses antihuman globulin to detect antibodies and/or complement present in a patient's plasma.	In the DAT, the patient's RBCs are washed and then incubated with antihuman globulin (AHG, Coombs reagent), which causes the RBCs to agglutinate if antibodies/complement are present. In the indirect antiglobulin test (indirect Coombs test), the patient's serum is incubated with RBCs with defined antigens; AHG is added, which causes agglutination if antibodies/complement are present. The DAT is useful in assessing patients with hemolytic anemia to exclude transfusion reactions and autoimmune and drug-related etiologies.	^33^	Coagulation
Endomysial antibody , IgA, serum	IMM 1.4	Negative	Celiac disease is an immune-mediated enteropathy of the small intestine triggered by gluten exposure in genetically susceptible individuals. IgA autoantibodies to the endomysium (connective tissue surrounding muscle cells) are elevated in 70–80% of patients with celiac disease or dermatitis herpetiformis. By comparison, anti-tissue transglutaminase autoantibodies have a sensitivity and specificity of 90–98% and 95 to 97%, respectively; therefore, this test is often the first-line test in celiac disease assessment.	This test is highly specific and can obviate the need for small bowel biopsies. Since this is an IgA antibody, the test cannot be used in individuals with IgA deficiency (about 2% of patients with celiac disease); in such patients, an IgG test can be performed. Titer generally correlates with disease severity and declines with strict adherence to a gluten-free diet.	^33^	Gastrointestinal
Eosinophil count	H 4.2	0.03–0.48 × 10^9^/L	Eosinophils arise from precursor cells in the bone marrow and are relatively infrequent in the peripheral blood in health. Eosinophil granules contain cytotoxic compounds, including major basic protein and eosinophil cationic protein. Eosinophils are capable of antigen presentation and are important in defense against parasitic infections.	Eosinophils are increased in parasitic infections, allergic conditions, asthma, drug hypersensitivity, autoimmune and connective tissue disorders, eosinophilic granulomatosis with polyangiitis, myeloproliferative neoplasms, and some types of lymphoma. Eosinophils are typically decreased in Cushing syndrome and corticosteroid therapy.		CBC
Erythrocyte sedimentation rate (ESR) aka Sed rate, sed, Westergren test, blood	IMM 1.1	Male 0–22 mm/h Female 0–29 mm/h	In health, the negatively charged red blood cell (RBC) membrane prevents aggregation. In the setting of inflammation, positively charged immunoglobulins and acute phase proteins (e.g., prothrombin, plasminogen, fibrin, C-reactive protein) bind to the RBC membrane, neutralizing the negative charge and causing RBCs to clump into stacks referred to as rouleaux. These large aggregates sediment more rapidly than individual RBCs, thereby increasing the ESR.	ESR can be elevated in infections, most anemias, inflammation (e.g., rheumatoid arthritis, SLE), pregnancy, malignancy (e.g., multiple myeloma, Waldenström macroglobulinemia, colorectal carcinoma, renal cell carcinoma); end-stage renal disease (uremia) and nephrotic syndrome (hypoalbuminemia). ESR is typically decreased in polycythemia vera (increased blood viscosity), sickle cell disease and hereditary spherocytosis.		Inflammatory
Erythropoietin (EPO), serum	CHEM 1.5	2.6–18.5 mIU/mL	EPO is a glycoprotein that is mainly produced by peritubular cells in the kidney after birth. This hormone stimulates red blood cell (RBC) production in the bone marrow.	Hypoxia stimulates EPO production. Decreased EPO production can be seen in renal failure while increased production is seen in hypoxemia (e.g., chronic obstructive pulmonary disease); the former can result in anemia and the latter in erythrocytosis. EPO levels are normal or decreased in polycythemia vera. Some neoplasms such as hepatocellular carcinoma, renal cell carcinoma, and adrenal adenoma may secrete erythropoietin.	^34^	Renal, Hematologic
Estradiol (E2), serum	CHEM 1.3	Varies with age, sex and sexual development (i.e., Tanner stage) Adult males: 10–40 pg/mL Adult females (premenopausal): 15–350 pg/mL Adult females (postmenopausal): <10 pg/mL	Estrone (E1), estradiol (E2), and estriol (E3) are three endogenously produced estrogens that are responsible for the development and regulation of the female reproductive system and secondary sex characteristics. Estrogens are produced from androgens such as testosterone through aromatization, an enzymatic step that may be blocked by aromatase inhibitor drugs. Estradiol is the dominant estrogen hormone present in nonpregnant, premenopausal females. It is produced in the ovarian follicles and regulates the menstrual cycle. It is produced in smaller amounts in the testes, adrenal glands and adipose tissue. After menopause, estradiol levels decrease dramatically. Increased estrogens, including estradiol are implicated as a driving factor in some malignancies including type 1 endometrial carcinomas and certain breast cancers.	Levels of estradiol and other estrogens vary depending on age, sex, menopausal status and stage of the menstrual cycle; therefore, clinical correlation is necessary. Evaluation of luteinizing hormone (LH) and follicle-stimulating hormone (FSH) levels can be useful. Levels rise during the follicular phase and peak during ovulation, falling again during the luteal phase. Estradiol levels are used to evaluate fertility, monitor ovulation and to evaluate oligomenorrhea and menopausal status.	^35^	Endocrine
Estrone (E1), serum	CHEM 1.3	Varies with age, sex and sexual development (i.e., Tanner stage) Adult males: 10–60 pg/mL Adult females (premenopausal): 17–200 pg/mL	Estrone (E1) is the dominant form of estrogen during menopause and is principally derived from peripheral aromatization of androstenedione in adipose tissue and the adrenal gland. Estrone acts as a precursor to estradiol, which is more potent than estrone, and conversion back and forth occurs.	Levels of estrone and other estrogens vary depending on age, sex, menopausal status and stage of the menstrual cycle; therefore, clinical correlation is necessary. E1 levels are used in conjunction with other steroid hormones to evaluate delayed/ precocious puberty (females > males), evaluation of sex steroid disorders (e.g., 17 alpha-hydroxylase deficiency) and in fracture risk assessment and monitoring hormone replacement therapy in postmenopausal women. Levels may be increased in the setting of hyperthyroidism, cirrhosis, Turner syndrome, estrogen- or androgen-producing tumors and polycystic ovary syndrome (PCOS).		Endocrine
Ethanol, blood	CHEM 1.7	Legal limit of intoxication in most of the United States: > 80 mg/dL Potentially lethal: ≥ 400 mg/dL	Ethanol is metabolized in the liver, primarily through an oxidative pathway in which alcohol dehydrogenase converts ethanol into acetaldehyde, which is further metabolized to acetic acid by aldehyde dehydrogenase. With increasing blood alcohol levels or chronic ethanol consumption, micro-somal cytochrome P450 metabolism of ethanol becomes increasingly significant, particularly the CYP2E1 isoform. CYP2E1 levels increase with chronic alcohol consumption, which contributes to increased tolerance.	Serum, plasma, and whole blood are appropriate samples for laboratory ethanol testing. When interpreting results, both serum and plasma ethanol levels are generally higher than whole blood due to its solubility in water (ratio of serum or plasma to whole blood ranges from 1.08 to 1.18). The blood ethanol test is most reliable < 8–12 h after ingestion. Whole blood ethanol concentrations used in the legal setting (e.g., driving under the influence) often uses % ethanol by volume where, for example, 80 g/dL is the same as 0.08%.		Toxicology
Factor IX (FIX) activity assay, plasma	H 2.1	65–140%	FIX is a protease that is part of the intrinsic coagulation pathway. It is activated by Factor XIa or Factor VIIa/tissue factor. In the presence of calcium, phospholipids and Factor VIIIa, FIXa activates Factor X, which generates thrombin from prothrombin. FIX is inhibited by anti-thrombin. The gene for FIX is on chromosome X.	Hemophilia B, also called Christmas disease, is an inherited deficiency of FIX and is an X-linked recessive disorder. It is much less common than hemophilia A (factor VIII deficiency). About one third of cases arise from spontaneous mutations and two thirds are inherited.	^36^	Coagulation
Factor V Leiden (FVL) mutation, blood	H 2.1	Negative	Factor V (FV) is a vitamin K-independent coagulation factor that is an essential cofactor in the conversion of prothrombin to thrombin. In FVL, a point mutation substitutes arginine for glutamine (R506Q) preventing cleavage by activated protein C (aPC); persistent Factor Va produces a hyper-coagulable state. This PCR-based test detects FV R506Q mutation.	FVL is the most common cause of inherited venous thromboembolism in individuals of European ancestry, though it can be seen in other groups due to population admixture. FVL increases the risk of thrombosis 25 to 50- fold in homozygotes and five-fold in heterozygotes; however, most heterozygotes do not develop blood clots. Compound heterozygotes with the prothrombin G20210A allele are not uncommon.		Coagulation
Factor VIII (FVIII) activity assay, plasma	H 2.1	55–200%	FVIII is a coagulation cofactor that is bound to and stabilized by von Willebrand factor (vWF) in the plasma. It is an essential cofactor in Factor X activation by Factor IX. This test measures the activity of FVIII in patient plasma and is reported as a percentage relative to reference normal plasma.	The FVIII gene is located on chromosome X. Hemophilia A is an X-linked recessive disorder due to an inherited deficiency of FVIII; it presents with hemarthroses and prolonged bleeding. Rare patients with homozygous von Willebrand disease may present with low FVIII levels and hemophilia-like bleeding. Autoantibodies to FVIII can inhibit its function, resulting in acquired hemophilia.		Coagulation
Ferritin, serum	H 3.2	Male: 24–336 μg/L Female: 11–307 μg/L	Ferritin is found in serum and in the cytoplasm of tissue macrophages; it is the major storage protein for iron. Ferritin concentration varies with age and sex and correlates with total iron stores; therefore, ferritin levels are low in iron-deficiency anemia and high in iron overload (e.g., hemo-chromatosis). Ferritin is an acute phase reactant and can be increased in acute and chronic inflammation as well as in chronic kidney disease and some malignancies.	Ferritin is often measured in combination with serum iron, transferrin saturation and total iron binding capacity; these tests may be less precise and do not distinguish depleted iron stores from impaired iron release (e.g., anemia of chronic inflammation). Low serum ferritin is highly specific for iron deficiency anemia. However, acute phase reaction can sometimes mask the low ferritin that would otherwise be seen in iron deficiency anemia.	^37^^,^^38^	Hepatic, Hematology
Fibrinogen, plasma	H 2.1	200–393 mg/dL	Fibrinogen (Factor I) is essential for formation of stable clots and thus hemostasis. Fibrinogen links activated platelets via the GpIIb-IIIa receptor and is cleaved by thrombin to form insoluble fibrin polymers. These polymers are further cross-linked by factor XIIIa to form a stable clot that is eventually broken down by plasmin.	Fibrinogen is an acute phase reactant synthesized by the liver and may be elevated in inflammatory conditions. Decreased levels may be due to underproduction (e.g., intrinsic liver disease, protein malnutrition, rare genetic disorders) or over “consumption” (e.g., disseminated intravascular coagulation).		Coagulation, Inflammatory
Folate, serum	HB 3.2	≥4.0 μg/L	Folate (vitamin B9) is an essential water-soluble vitamin that is a coenzyme for one carbon metabolism. Biochemically, a carbon unit from serine or glycine is transferred to tetrahydrofolate (THF) to form methylene-THF, which then is 1) used in the synthesis of thymidine (and incorporation into DNA); 2) oxidized to formyl-THF for use in the synthesis of purines (precursors of RNA and DNA); or 3) reduced to methyl-THF, which is necessary for the methylation of homocysteine to methionine.	Megaloblastic anemia (characterized by large, abnormally nucleated erythrocytes in the bone marrow) is the major clinical manifestation of folate deficiency. Low serum folate concentrations in pregnancy are associated with neural tube defects. Folate deficiency may be due to poor absorption (e.g., celiac disease), insufficient intake (e.g., chronic excess alcohol use) and medications (e.g., methotrexate). Folate deficiency in the United States has become less common with widespread supplementation of breads, grains, and other foods with folate.		Hematologic
Follicle-stimulating hormone (FSH), serum	CHEM 1.3	Varies with age, sex, menstrual cycle and sexual development (i.e., Tanner stage) Adult males: 1.2–15.8 IU/L Adult females: Premenopausal: Follicular: 2.9–14.6 IU/L Midcycle: 4.7–23.2 IU/L Luteal: 1.4–8.9 IU/L Postmenopausal: 16.0–157.0 IU/L	FSH is a gonadotropin released by the anterior pituitary in response to gonadotropin releasing hormone (GnRH). It is most specifically associated with initiating follicular growth in ovaries. The mid-menstrual cycle surge in FSH and luteinizing hormone (LH) culminates in ovulation.	FSH assays can be useful in assessing fertility, in the evaluation of menstrual irregularities, predicting ovulation and investigating pituitary disorders. FSH and LH may be elevated in primary gonadal failure, precocious puberty and menopause. FSH may be normal or decreased in polycystic ovary syndrome. Both FSH and LH are decreased with pituitary or hypothalamic dysfunction.		Endocrine
Gamma-glutamyltranspeptidase (GGT), serum	CHEM 1.4	Adult males: 8–61 U/L Adult females: 5–36 U/L	GGT is a membrane protein that transfers gamma glutamyl groups from substrates such as glutathione to other peptides and amino acids. It is central to the synthesis and degradation of glutathione, an antioxidant that also plays a role in the detoxification of xenobiotics. GGT is present in multiple tissues including liver, kidney and pancreas.	The highest levels of GGT elevations are seen in intra- and posthepatic biliary obstruction; moderate levels are less specific and can be seen in all types of liver disease (e.g., alcohol-related hepatitis) and with some medications (e.g., anticonvulsants, oral contraceptives). Combined elevations of alkaline phosphatase and GGT suggest biliary tract disease.	^39^	Hepatic
Glucose, serum	CHEM 1.3	≥1 year old: 70–140 mg/dL	Physiologic glucose levels are primarily maintained by insulin and glucagon. Hyperglycemia may be due to either insufficient insulin (e.g., type I diabetes) or peripheral insulin resistance (e.g., type II diabetes). Measurement of serum glucose is useful in the diagnosis and management of diabetes.	Glucose levels vary depending on whether or not an individual is fasting. For the diagnosis of diabetes, a repeat test on a different day is necessary. Hemoglobin A1c provides a longer-term assessment of glucose control and is thus complementary to day-to-day glucose levels. Hypoglycemia, usually in the setting of excess insulin dose, can be life threatening.		BMP/Chem 7, Endocrine
Growth hormone (GH; somatotropin), serum	CHEM 1.3	Adult males: 0.01–0.97 ng/mL Adult females: 0.01–3.61 ng/mL	GH secretion from the somatotroph cells in the anterior pituitary is stimulated by ghrelin (stomach) and GH releasing factor (GHRH) (hypothalamus) and inhibited by somatostatin (hypothalamus). GH and insulin-like growth factor 1 (IGF-1) inhibit GH secretion. GH induces growth in most tissues and organs, but its effect is most pronounced on cartilage and bone. GH levels increase in childhood, are at their peak during puberty and decrease with increasing age.	Low levels of GH in infancy or early childhood can cause dwarfism and elevated levels causes gigantism in children (before physis closure) or acromegaly once the growth plate has closed. GH is released in a pulsatile fashion such that random GH levels are of little diagnostic value. GH stimulation tests use drugs (e.g., l-dopa, clonidine) to prompt GH secretion. IGF-1 levels are also informative. Since GH counteracts the effect of insulin, type 2 diabetes and hyperlipidemia are associated with acromegaly.		Endocrine
Growth hormone releasing hormone (GHRH), serum	CHEM 1.3	Baseline ranges: 5–18 pg/mL	GHRH stimulates synthesis and secretion of growth hormone (GH) by the anterior pituitary. GHRH is secreted in a pulsatile fashion, with a bolus at the onset of sleep. Hypothalamic somatostatin suppresses release of both GH and GHRH. GHRH synthesis is inhibited by GH and IGF-1. GHRH elaboration is modulated by a number of proteins including thyroid hormones, glucocorticoids, leptin, ghrelin and testosterone and estrogen.	Excess GHRH may be due to hypothalamic tumors or as a paraneoplastic syndrome (e.g., well differentiated neuroendocrine tumors). Excess GHRH can cause gigantism or acromegaly, sporadically or as part of multiple endocrine neoplasia syndrome. Decreased GHRH can result in dwarfism.		Endocrine
Haptoglobin, serum	H 4.1	30–200 mg/dL	Haptoglobin is a serum protein produced by the liver that binds to hemoglobin released by lysed red blood cells (RBCs). Hemoglobin-haptoglobin complexes are rapidly removed from the circulation by the reticuloendothelial system. If the rate of hemolysis overwhelms the binding capacity of serum haptoglobin, free hemoglobin passes through the kidneys (hemoglobinuria). Haptoglobin is an acute-phase reactant and its levels may increase in certain conditions (e.g., extensive burns).	Haptoglobin levels are useful in the evaluation of hemolysis. Haptoglobin levels decrease in extensive hemolysis (e.g., autoimmune hemolytic anemia, transfusion reaction) and may be persistently low with chronic intravascular hemolysis. Significant liver disease (e.g., cirrhosis) can also cause reduced haptoglobin levels. Haptoglobin elevations can be seen during stress or inflammation; comparison with additional acute-phase reactants (e.g., CRP) may be indicated.		Hematologic
*Helicobacter pylori* breath test	M 1.3	Negative	*H. pylori* causes chronic gastritis, peptic ulcer disease, gastric adenocarcinoma and lymphoma. The organism produces urease, which neutralizes gastric acid and provides ammonia for bacterial protein synthesis. Patients with suspected *H. pylori* infection ingest a small amount of urea labeled with a rare isotope (e.g., nonradioactive carbon-13); when *H. pylori* organisms are present, they split the urea, releasing isotope-labeled carbon dioxide in the patient's breath. The carbon isotope can be quantified and is proportional to the urease activity.	This high sensitivity, high specificity test may be used for initial diagnosis of *H. pylori* infection and for confirming *H. pylori* eradication. By comparison, since infection may be localized, gastric biopsy may provide a false negative. Antibiotics and drugs that suppress gastric acid production may also cause false negative results. False positive results may be seen in the setting of achlorhydria and infection with other urease-positive organisms. This test should not be used for screening asymptomatic patients.	^24^	Gastrointestinal
*Helicobacter pylori* stool test	M 1.3	Negative	*H. pylori* causes chronic gastritis, peptic ulcer disease, gastric adenocarcinoma and lymphoma. *H. pylori* is shed in the stool of infected patients. Two types of tests evaluate the presence of *H. pylori* in the stool: 1) enzyme immunoassay or immunochromatography for bacterial antigens and 2) PCR for *H. pylori* bacterial sequences. These tests are noninvasive, in contrast to gastric biopsy.	These high sensitivity, high specificity tests may be used for initial diagnosis of *H. pylori* infection and for confirming *H. pylori* eradication. Effective treatment results in loss of stool antigen within 4+ weeks. Antibiotics and drugs that suppress gastric acid production may cause false negative results.	^24^	Gastrointestinal
Hematocrit (Hct), blood	H 4.2	Males: 38–49% Females: 35%–45%	Spun hematocrit (packed cell volume) is the percentage of blood volume that is taken up by packed red blood cells (RBCs) in a centrifuged sample. Hematology analyzers calculate hematocrit from the mean corpuscular volume (MCV) and the red blood cell count (Hct = (MCV x red blood cell count/10)) or derive it by measuring the cumulative volume of red blood cells in the sample.	The hematocrit is decreased in anemia and increased in polycythemia. It may be falsely elevated in the setting of sickled RBCs, dehydration and severe hypertriglyceridemia and may be falsely decreased following volume resuscitation. The hematocrit is approximately three times the hemoglobin (assuming the RBCs are normal in size and shape).	^40^	Hematologic
Hemoglobin A1c (HbA1c), blood	CHEM 1.3	4.0–5.6%	When glucose attaches nonezymatically to hemoglobin, a glycated hemoglobin (HbA1c) is formed. This process occurs continually and therefore reflects mean plasma glucose levels over the course of red blood cell's life span of 8–12 weeks. Patients with high average blood concentrations of glucose (e.g., diabetes) will have higher HbA1c levels than those with unimpaired glucose metabolism.	HbA1c is the key laboratory test for monitoring long-term glucose control. HbA1c is also a diagnostic test for diabetes: HbA1c of 6.5% or higher on two different days is diagnostic of diabetes. HbA1c can also identify patients who may become diabetic: values of 5.7–6.4% are associated with increased risk of diabetes. HbA1c may be falsely low in conditions associated with shortened red blood cell lifespan (e.g., hemolysis, blood loss, hemoglobinopathies).		Endocrine
Hemoglobin S (HbS), blood	H 4.4	Absent	HbS has a valine residue instead of the normal glutamate residue at position 6 of beta-globin. The protein tends to aggregate and polymerize in the deoxygenated state. Homozygosity for HbS results in sickle cell anemia (SCA), while heterozygosity for this allele results in sickle cell trait, which is typically asymptomatic. The HbS allele confers a heterozygote advantage in the setting of falciparum malaria and proliferated in ancestral populations affected by this parasite (e.g., sub-Saharan Africa, parts of Southern Europe, the Middle East and India).	Recent transfusions can lower HbS concentration and complicate diagnosis of sickle cell disease. Measurement of HbS pre- and post-transfusion by serum electrophoresis is frequently used to monitor patients with SCA on regular transfusion protocols. In sickle cell trait, HbS should be between 35% and 45%; higher values suggest concurrent beta thalassemia trait, while lower values suggest concurrent alpha thalassemia trait. The presence of HbS may artifactually alter hemoglobin A1c levels.		Hematologic
Hemoglobin, blood	H 4.2	Males: 13.2–16.6 g/dL Females: 11.6–15.0 g/dL	Hemoglobin is the oxygen-carrying molecule in red blood cells (RBCs). The hemoglobin molecule is a tetramer that, after the first year of life, consists of two alpha globin chains and two beta globin chains. Each subunit contains a heme molecule composed of an iron molecule in a porphyrin ring. Each heme molecule can bind one oxygen molecule. Automated hematology analyzers calculate hemoglobin by lysing the RBCs and measuring the hemoglobin concentration by optical density.	Hemoglobin is decreased in anemia and increased in polycythemia. It can be falsely elevated in hyperlipidemia due to increased plasma turbidity. Hemoglobin is approximately one third the hematocrit (assuming the RBCs are normal size and shape).	^40^^,^^41^	Hematologic, CBC
Heparin-PF4 IgG antibody, serum	H 2.1	Absent	Antibodies to heparin-platelet factor 4 (PF4) complexes form in a subset of patients following heparin administration and cause heparin-induced thrombocytopenia (HIT), which is characterized by a decrease in platelet count of 50% or more from baseline, generally beginning 5–10 days after heparin administration. These patients are at risk for venous and arterial thromboembolism.	While the test is sensitive (98–100%), specificity is limited since not all anti-PF4 antibodies activate or deplete platelets. Assays that specifically measure heparin-PF4 IgG antibodies are more sensitive and specific than those that also measure IgA and IgM antibodies. In most laboratories, measurement is by ELISA. The serotonin release assay is more sensitive and is recommended for confirmation of a positive result.	^42^	Coagulation
Hepatitis A virus (HAV) IgG antibody, serum	M 3.1	Negative	HAV is a picornavirus that is spread by the fecal-oral route. IgG antibodies directed against HAV (IgG anti-HAV) are produced at the onset of clinical symptoms. These antibodies persist and provide immunity for the patient's lifetime.	IgG anti-HAV are produced by either acute infection with hepatitis A or through immunization. A positive test indicates immunity to HAV but does not indicate acute infection.	^43^	Hepatic
Hepatitis A virus (HAV) IgM antibody, serum	M 3.1	Negative	HAV is a picornavirus that is spread by the fecal-oral route. IgM antibodies directed against HAV (IgM anti-HAV) are produced 5–10 days before the onset of clinical symptoms; levels decline after 3–6 months and become undetectable.	The presence of IgM anti-HAV is used to diagnose acute infection with HAV. The total anti-HAV test assesses both IgG and IgM antibodies. HAV infection is associated with acute, self-limited illness and does not lead to a carrier state or to chronic disease.	^43^^,^^44^	Hepatic
Hepatitis A virus (HAV) polymerase chain reaction (PCR), serum	M 3.1	Negative	PCR can be used to detect HAV RNA during the viremic period, shortly after infection, until alanine aminotransferase (ALT) levels decline.	Detection of viral RNA can be used to confirm acute infection and is particularly useful in assessing outbreaks or response to therapy. It can also be used to confirm that blood products are free of virus prior to transfusion. This test is not as commonly used as serologic tests for diagnosis in clinical practice.	^43^^,^^44^	Hepatic
Hepatitis B virus core (HBc) antibody, IgM, serum	M 3.1	Negative	HBc is a nucleocapsid protein necessary for virion assembly. IgM antibodies to HBc can be detected after symptom onset and remain in the serum for about 6 months after initial infection. A positive test for IgM indicates recent infection. HBc IgM may be the only serologic test that is positive once HBV surface antigen declines and before the appearance of hepatitis B virus (HBV) surface antibody (“serologic window period”).	Anti-HBc IgM is produced during acute infection and decreases after a few months, regardless of whether the infection is acute and resolves or remains chronic. Anti-HBc antibodies are produced only when the patient has been naturally infected with hepatitis B and are not seen in individuals who have been vaccinated against HBV.		Hepatic
Hepatitis B virus e-antibody (HBeAb), serum	M 3.1	Negative	HBeAb is seen in patients recovering from acute hepatitis and is typically present before HBsAg to HBsAb conversion. As HBeAb increases, HBeAg decreases.	HBeAb is a sign of resolving acute hepatitis. HBeAb is produced in patients who have been naturally infected with hepatitis B and is absent in individuals who have been vaccinated.		Hepatic
Hepatitis B virus surface antibody (HBsAb), serum	M 3.1	Negative	HBsAb are antibodies to the 3 related viral envelope proteins encompassed by HBsAg. Levels typically increase with resolution of acute hepatitis and falling HBsAg. However, in some patients, HBsAb is not detectable for months after HBsAg disappears; in such patients, diagnosis can be confirmed by IgM for hepatitis B virus core protein.	HBsAb will be present after vaccination or after clearance of active infection, but may be delayed for months. Once present, it persists for life. HBsAb can be seen in vaccinated individuals (since HBsAg is the basis for the vaccine) or in individuals who have been naturally exposed to HBV.		Hepatic
Hepatitis B virus surface antigen (HBsAg), serum	M 3.1	Negative	HBsAg encompasses 3 related envelope glycoproteins (large, medium and small). Large HBsAg is seen in complete, infective virions whereas small HBsAg are noninfective. HBsAg is the first serologic marker to be detectable, even before a patient is symptomatic, typically 6–16 weeks after HBV infection. With resolution of acute hepatitis, HBsAg disappears about 12 weeks after symptom onset.	Persistence of HBsAg 6 months or more is seen in chronic carrier states and chronic HBV infection. Recombinant HBsAg is the basis for the HBV vaccine. Therefore, HBsAg may be seen transiently in individuals (particularly neonates and children) who have recently received the HBV vaccine; typically, HBsAg is only seen in patients who have been naturally infected with hepatitis B.		Hepatic
Hepatitis B virus core (HBc) total antibodies, serum	M 3.1	Negative	HBc is a nucleocapsid protein necessary for virion assembly. Antibodies to HBc can be detected soon after symptom onset and after antibodies to HBV surface antigen are present. Initial antibodies are IgM, followed by IgG. Total Anti-HBc antibody is a measure of IgM and IgG.	Total HBc antibody persists for life; therefore, a positive test does not indicate recent or chronic infection. Neonates with a positive test result should be evaluated for anti-HBc IgM to exclude transplacental antibody transfer from the mother. Anti-HBc antibodies are produced only when the patient has been naturally infected with hepatitis B and are not seen in individuals who have been vaccinated against HBV.		Hepatic
Hepatitis B virus DNA PCR, serum	M 3.1	Negative	HBV DNA is detectable by 30 days after infection, about 3 weeks before HBsAg appears, peaks with acute hepatitis, and slowly declines with resolution of infection. Although serologic methods are the primary means of diagnosis in acute HBV infection, HBV DNA PCR is useful for the diagnosis of early infection, prior to appearance of HBsAg; differentiating between active and inactive HBV infection; and monitoring response to anti-HBV treatment.	HBV DNA levels can be useful in the further classification of patients with chronic HBV infection as chronic active (high levels of HBV DNA, positive for HBeAg) and chronic inactive (low or no HBV DNA, negative for HBeAg). With reactivation of inactive chronic HBV, HBeAg may be negative, leaving HBV DNA as the only indication of active viral replication.		Hepatic
Hepatitis B virus e-antigen (HBe–Ag), serum	M 3.1	Negative	The hepatitis B virus e-antigen (HBeAg) is a secretory protein that is seen in active viral replication. HBeAg can be detected soon after HBV surface antigen appears.	While HBeAg is detectable, active infection is ongoing. It can be seen in both acute and chronic hepatitis. Some strains of hepatitis B do not produce HBe antigen, however. High HBeAg levels in the absence of HBe antibody (HBeAb) are associated with active viral replication and high infectivity; as HBeAb levels rise, infectivity decreases. HBeAg may be used to monitor disease activity in individuals who are carriers for HBV and individuals with chronic hepatitis B infection.		Hepatic
Hepatitis C virus (HCV) antibody screen, serum	M 3.1	Negative	IgG antibodies to HCV are generally not detectable for the first two months after infection, but are typically seen by 6 months. Delay is more common in individuals who are immunocompromised. Antibodies do not confer protection from the virus. Though typically persistent, they can be lost over time.	The majority of patients infected with HCV are asymptomatic. Screening for HCV is recommended at least once in adults and during each pregnancy. Additional testing may be needed with certain medical conditions, exposures, or risk factors. A negative antibody screen is seen in truly negative individuals but can also be seen in very early infection (in the window period before antibodies are detectable). Since IgG antibodies typically persist, a positive test requires RNA testing to confirm active disease.	^43^	Hepatic
Hepatitis C virus RNA detection by reverse transcription polymerase chain reaction (RT-PCR), serum	M 3.1	Undetected	Hepatitis C virus (HCV) is a small, enveloped RNA virus; its RNA is detectable 1–3 weeks after infection (1–1.5 months before HCV antibodies are seen) and can be reported either qualitatively or quantitatively (via real time RT-PCR).	The majority of patients infected with HCV are asymptomatic. Serologic testing is the usual modality for determining HCV infection. In chronic HCV infection, circulating HCV RNA persists in 90% of patients despite the presence of neutralizing antibodies. Viral load (quantitative HCV RNA) and genotype are used to guide treatment decisions; viral load is useful in following response to treatment. A positive antibody screen with no detectable HCV RNA indicates clearance of the infection.	^43^	Hepatic
Hepatitis D virus total antibodies, serum	M 3.1	Negative	Hepatitis D virus (HDV) is a defective RNA virus that cannot replicate without HBV, which provides HBsAg. Infection can either by simultaneous with HBV (coinfection) or acutely in the context of chronic HBV infection (superinfection). IgM anti-HDV appears 2–3 weeks after infection and is a reliable indicator of recent HDV exposure, but is frequently short-lived. Acute coinfection by HDV and HBV is associated with the presence of IgM against HDAg and HBcAg (denoting new infection with hepatitis B). When chronic hepatitis arises from HDV superinfection, HBsAg is present in serum, and anti-HDV antibodies (IgG and IgM) persist for months or longer.	Patients with HDV antibodies should be evaluated for IgM HBc; their presence indicates HBV-HDV coinfection, which has a higher risk for progression to cirrhosis. A negative IgM HBc indicates superinfection in a patient with chronic HBV.		Hepatic
Hepatitis E Virus IgM and IgG antibodies, serum	M 3.1	Negative	Hepatitis E virus (HEV) is an unenveloped RNA virus that is primarily transmitted by the fecal-oral route. Like HAV, HEV causes acute, self-limited illness; however chronic hepatitis can occur in patients who are immunocompromised. Symptoms typically become apparent 2 weeks to 2 months after exposure. IgM antibodies are detectable early and disappear within 4–5 months. IgG antibodies appear almost immediately after the IgM response; it is not clear how long they persist.	Acute HEV infection in the third trimester of pregnancy has a high mortality rate (15–25%). Patients who are immunocompromised may have prolonged viral shedding. Tests for IgM and IgG antibodies may be negative, even in patients with HEV infection, but infection can be confirmed by PCR and stool culture.	^43^	Hepatic
Hexoasaminidase A activity, leukocytes	ACD 3.2	≥16 years old: 56–80% of total hexosaminidase activity	Hexosaminidase A levels are decreased in the lysosomal storage disorder, Tay-Sachs disease, a GM2 gangliosidosis caused by deficiency of the ∝ subunit of hexosaminidase A. Loss of this enzyme causes GM2 ganglioside to accumulate in cells. Hexosaminidase A and hexosaminidase B are isoenzymes. This test measures activity of total hexosaminidase (A and B) using an artificial substrate, followed by heat inactivation of hexosaminidase A and a repeat activity test to calculate the percentage activity of hexosaminidase A.	False positive results can be seen due to pseudodeficiency alleles that produce enzymes that are active in vivo but that have decreased activity with the artificial substrate used in the enzyme assay. This test can also be used to detect carrier status.	^45^^,^^46^	Nervous
High-risk HPV (hrHPV) test, site varies	GE 3.1	Not detected	Most invasive cervical cancers are squamous cell carcinoma; persistent infection with high-risk human papillomavirus (HPV) is usually necessary but not sufficient for the development of squamous cell carcinoma. HrHPV DNA integrates into the host genome and disrupts the viral DNA in the E1/E2 open reading frame, leading to loss of the E2 viral repressor and over-expression of E6 and E7 oncoproteins. E6 and E7 normally inactivate tumor suppressors, activate cyclins and inhibit apoptosis; therefore, overexpression of E6 and E7 extends the lifespan of epithelial cells. E6 and E7 bind to and increase the degradation of p53 and Rb tumor suppressor proteins respectively, and E7 inactivates the cyclin-dependent kinase (CDK) inhibitor p21.	The goal of HPV screening is to identify HPV types likely to progress to cervical carcinoma. Pap test and hrHPV tests may be used alone or in combination; many countries and professional organizations recommend testing for hrHPV types first, followed by a Pap test in some situations.	^47^	Gynecologic
High density lipoprotein (HDL), serum	CHEM 1.2	Males: ≥ 40 mg/dL Females: ≥ 50 mg/dL	Of the lipoproteins (HDL, low density lipoprotein [LDL], very low density lipoprotein [ VLDL]), HDL is the smallest and has the highest ratio of proteins to lipids (about 50% protein). HDL transports cholesterol from the periphery to the liver where it is catabolized and excreted.	Low levels of HDL are a risk factor for atherosclerosis. HDL levels may be increased due to exercise, alcohol consumption and some medications (e.g., hormone replacement therapy).		Vascular
HIV antibody/antigen, plasma	M 3.4	Negative	Third generation IgM and IgG tests can detect the presence of antibodies 20–30 days after human immunodeficiency virus (HIV) exposure and are often the initial screening test used for HIV infection. Combination HIV antigen/antibody tests detect IgG and IgM antibodies as well as the HIV p24 antigen and can become positive 15–20 days after exposure. By comparison, HIV RNA tests can be positive 10–15 days after exposure.	First-time positive tests should be confirmed by another method (e.g., HIV RNA, HIV-1/HIV-2 differentiation assay). Combination tests can detect p24 antigen before seroconversion (the “window period”). Detection occurs earlier for both antibody tests and combination tests with blood taken by venipuncture rather than from a finger stick (18–45 days vs 18–90 days). Rapid antibody tests have > 99% specificity and sensitivity in chronic infection, but are less sensitive in acute infection.		Immunologic
HIV DNA, plasma	M 3.4	Undetected	Diagnosis of HIV infection is primarily serologic (i.e., detection of HIV-specific antibodies). However, in neonates (who have immature immune systems and who may have maternal anti-HIV antibodies), in early HIV infection (< 30 days from exposure) or in individuals with equivocal serologic results, assays for HIV pro-viral DNA and HIV RNA are useful adjuncts. These tests are typically informative 10–14 days after infection.	Although antiretroviral therapy can decrease viral load in plasma to undetected levels, disease is not eradicated. Viral rebound is linked to the persistence and transcriptional activation of HIV proviral DNA, which can be assessed by this test. Neonates born to mothers who are HIV positive should have DNA/RNA tests at multiple intervals (e.g., 0–2 days, 2 weeks, 1–2 months, and 4–6 months after birth). For accurate diagnosis in children under two years, two consecutive positive nucleic acid tests are necessary.		Immunologic
HIV NAT (nucleic acid test), plasma	M 3.4	Undetected	NAT is used to detect a particular nucleic acid sequence for a specific organism (e.g., HIV, *Neisseria gonorrhoeae*). Quantitative assays can be used to guide antiretroviral therapy, while qualitative assays are used for HIV diagnosis. NAT can detect infection 10–33 days after exposure, compared to antigen/antibody tests (18–45 days after exposure) and antibody tests (23–90 days). NAT is based on DNA or RNA detection using RT-PCR, nucleic acid sequence-based amplification (NASBA) and technologies based on branched chain DNA methods (b-DNA), transcription-mediated amplification (TMA) and real time PCR.	The NAT qualitative test is mainly used at donor centers to confirm safety of blood products and for early diagnosis in infants born to mothers who are HIV positive.	^48^	Immunologic
HIV RNA (HIV viral load), plasma	M 3.4	Undetected	More than 99% of HIV cases worldwide are due to HIV-1. Therefore, this is the viral type most commonly assessed. HIV viral load (number of HIV-1 RNA copies/mL of plasma) correlates with disease stage and, combined with CD4^+^ T cell numbers, is useful in monitoring response to treatment. This test determines how actively virus is replicating in a person with HIV infection. US Health and Human Services defines > 200 copies/mL as virologic failure.	When 2 tests performed at least 2–4 weeks apart show virologic failure, drug-drug interactions and patient adherence to the regimen should be evaluated. At determined levels (e.g., 500 copies/mL), testing for drug resistant genotypes may be warranted.		Immunologic
Human chorionic gonadotropin (hCG), serum	CHEM 1.3	Males and non-pregnant females: < 5 mIU/mL Varies during pregnancy	hCG is a hormone comprised of α and β subunits. The α subunit is the same as that of follicle stimulating hormone (FSH), luteinizing hormone (LH) and thyroid stimulating hormone (TSH); therefore, most tests assess levels of the β subunit to increase sensitivity. During the first trimester, hCG synthesized by placental syncytiotrophoblastic cells stimulates the corpus luteum to secrete progesterone; subsequently, the placenta secretes progesterone and hCG levels fall. hCG may also be secreted by neoplasms derived from germ cell, placental, or embryonic origins.	hCG is commonly measured in pregnancy tests and is usually significantly above baseline levels by 4–5 weeks gestation in a healthy uterine pregnancy. hCG rises more slowly in ectopic pregnancy. hCG levels may be extremely elevated in choriocarcinoma and molar pregnancies. In males, elevated hCG raises the possibility of seminomatous and nonseminomatous testicular tumors. hCG is clinically useful as a tumor marker for diagnosis and disease monitoring.		Endocrine
Human herpesvirus 8 (HHV8), blood	M 3.3	Quantitative real-time PCR: <1000 copies/mL	HHV8 (also known as Kaposi sarcoma-associated herpesvirus, KSHV) is a DNA virus that is associated with Kaposi sarcoma (KS), primary effusion lymphoma and Castleman disease. HHV8 seroprevalence varies geographically and is common, underscoring the importance of host factors (e.g., immune status, proinflammatory cytokines) in development of disease. HHV8 infects multiple cell types, including B cells and endothelial cells, in which it can remain latent, thereby escaping immune surveillance and resulting in lifelong infection. When HHV8 enters the lytic phase, intact, infectious virions are produced.	HHV8 is associated with all four types of KS (i.e., classic, endemic, organ transplant-associated, epidemic/AIDS related). KS will often regress with reduction of immunosuppressive medication in transplant patients; however, this raises the risk of transplant rejection. Primary effusion lymphoma arises in the pericardial, pleural and peritoneal cavities. HHV8 positivity can be seen in all variants of Castleman disease in patients who are HIV-positive and patients who are HIV-negative.		Vascular
International normalized ratio (INR), plasma	H 2.1	0.9–1.1	The prothrombin time (PT), which is used as a screening test for coagulopathies, shows intra- and interlaboratory variation due to differences in instruments used and variable tissue factor activity. To correct for these differences, the INR is calculated by dividing the patient's PT by a control PT with a standardized thromboplastin reagent.	INR is most commonly used as a means to monitor response in patients who are being anticoagulated with vitamin K antagonists (e.g., warfarin). It is also used to assess patients with bleeding diatheses secondary to defects in the extrinsic pathway, in disseminated intravascular coagulation and to monitor patients with end-stage liver disease.		Coagulation
Intrinsic factor (IF) antibodies, serum	IMM 1.4 and H 4.1	Negative	Intrinsic factor is secreted by gastric parietal cells and binds to vitamin B_12_, facilitating its absorption in the terminal ileum. In pernicious anemia, autoantibodies to IF prevent vitamin B_12_ binding, leading to vitamin B_12_ deficiency. Vitamin B_12_ deficiency can manifest as megaloblastic anemia and neurologic symptoms.	Although anti-IF antibodies are very specific, they are positive in only about 50% of patients with pernicious anemia. False positives can occur when patients have received intramuscular or subcutaneous vitamin B_12_.		Gastrointestinal
Iron, serum	H 3.2	Males: 50–150 mg/dL Females: 35–145 mg/dL	Most of the body's iron is incorporated into hemoglobin. Iron can also be stored in tissues (complexed with ferritin) or transported in the serum (bound to transferrin). When iron stores in the body are depleted (e.g., iron deficiency anemia), transferrin levels increase in the blood, which increases the total iron binding capacity (TIBC). Percent saturation is calculated using serum iron and TIBC. Iron stores can also be evaluated via bone marrow aspirate.	In iron deficiency, TIBC is increased, since transferrin levels are relatively high compared to iron content; in iron overload, TIBC decreases since the free transferrin diminishes. TIBC, serum iron and percent saturation are often assessed in the setting of iron deficiency anemia; however, serum ferritin is more sensitive and more accurately reflects the body's iron stores.		Hematologic
Lactate dehydrogenase (LDH), serum	CHEM 1.4	≥ 18 years: 122–222 U/L	Lactate dehydrogenase is an enzyme that is present in almost all cells. Five different isomers of LDH are expressed at different levels in different tissues. High concentrations of LDH are present in liver, muscle, and kidney; moderate concentrations are present in red blood cells.	Serum LDH levels are increased in conditions associated with cell damage/death, including myocardial infarction, liver disease, hemolytic anemia, megaloblastic anemia, pulmonary embolus, and tumors such as metastatic melanoma and lymphoma. Falsely elevated serum LDH levels may occur if the blood specimen becomes hemolyzed in vitro.		Hepatic
Lead, blood	CHEM 1.7	Children <5 mg/dL Adults: ≤ 40 mg/dL (occupational)	Most lead is absorbed via the gastrointestinal tract and can be distributed throughout the body, with some preference to bone, erythrocytes and keratin-rich tissues. Adults typically absorb 1–10% of the ingested amount; this increases to up to 50% in children, particularly if they have coexistent nutritional deficiencies. Lead forms covalent bonds with protein cysteine sulfhydryl groups; this contributes to renal toxicity and accumulation in keratin-rich hair. Lead decreases heme biosynthesis and acts as a mitochondrial toxin.	Clinical manifestations vary by age. Children may show decreased nerve conduction velocity, decreased vitamin D metabolism, abdominal pain, anemia, nephropathy and encephalopathy. Lead incorporated into the physes can be seen radiographically as “lead lines”. In adults, initial presentation is typically elevations of systolic pressure and decreased hearing, progressing to peripheral neuropathies and nephropathy, and finally to anemia and encephalopathy at later stages.	^26^	Toxicology
Lipase, serum	CHEM 1.2	13–60 U/L	Lipase is a digestive enzyme produced in pancreatic acinar cells and secreted into the duodenum to digest lipids. With acinar cell injury (e.g., acute pancreatitis), lipase can be released into the pancreas itself where it contributes to local tissue damage, including acute inflammation, autodigestion of pancreatic parenchyma, fat necrosis and vascular damage.	Serum lipase is elevated in acute pancreatitis and is a more sensitive and specific test than serum amylase. In this setting, serum lipase is typically elevated > three times the upper limit of normal. The degree of lipase elevation does not correlate with the severity of pancreatitis. Serum lipase increases within 4–8 h in acute pancreatitis and may remain elevated for up to 14 days. Lipase has limited utility for the diagnosis of chronic pancreatitis due to destruction of pancreatic parenchyma.		Pancreatic
Lipoprotein (a) (Lp[a]), serum	CHEM 1.2	<5 mg/dL	Lp(a) is composed of apolipoprotein(a) bound to the apo(b) moiety of LDL via a disulfide bridge. Lp(a) is atherogenic and prothrombotic. Proposed mechanisms include interference with fibrinolysis, macrophage binding and recruitment in atherosclerotic plaques, and disruption of normal endothelial function.	Increased lipoprotein (a) is an independent risk factor for cardiovascular disease		Vascular
Low density lipoprotein (LDL), serum	CHEM 1.2	Adults: <100 mg/dL, desirable	Fifty percent of the total lipoprotein mass is LDL which is the product of VLDL metabolism. It is composed of cholesterol (50%), protein (25%), phospholipid (20%) and a trace amount of triglycerides (TG). LDL delivers cholesterol to peripheral tissues: ApoB located on the LDL surface binds to LDL receptors on the cell surface. LDL in serum can be measured directly but more commonly is estimated by the Friedewald equation: [LDL] = [Total cholesterol] - [HDL] - ([TG]/5), with concentrations in mg/dL. The calculated value may be inaccurate in the setting of markedly elevated triglycerides.	LDL is a major component of atheromatous plaques and elevated LDL is a risk factor in cardiovascular disease. Serum levels are affected by lifestyle factors (e.g., diet, exercise) and some diseases. Conditions in which LDL levels are elevated include familial hypercholesterolemia, hypothyroidism, uncontrolled diabetes, nephrotic syndrome, Cushing syndrome, and corticosteroid use. LDL levels are typically decreased in severe liver disease, hyperthyroidism and in the setting of severe acute or chronic illness, malnutrition, malabsorption or extensive burns.		Vascular
Luteinizing hormone, serum	CHEM 1.3	Males: > 18 years: 1.3–9.6 IU/L Females: Premenopausal: Follicular: 1.9–14.6 IU/L Midcycle: 12.2–118.0 IU/L Luteal: 0.7–12.9 IU/L Postmenopausal: 5.3–65.4 IU/L	Luteinizing hormone (LH) is a hormone co-secreted with follicle stimulating hormone (FSH) by the anterior pituitary gland in response to gonadotropin-releasing hormone (GnRH) from the hypothalamus. It is composed of α and β subunits. The α subunit is identical with the α subunit of follicle stimulating hormone (FSH), thyroid stimulating hormone, and human chorionic gonadotropin. LH is essential for reproduction in both sexes.	LH is measured in the workup of hypogonadism and will be low if failure is central (pituitary gland or hypothalamus) and elevated if failure is primary due to the ovaries or testes. LH is measured to predict ovulation and in the workup of menstrual irregularities and infertility.		Endocrine
Lymphocyte count, blood	H 4.2	0.95–3.07 × 10^9^/L	Lymphocytes are a subset of white blood cells (WBC) that includes T cells, B cells, and NK cells. Lymphocytes are the most plentiful circulating WBC type in young children, and the second most plentiful WBC type (after neutrophils) in healthy adults.	The most important causes of significant lymphocytosis include viral infections, autoimmune diseases and lymphoproliferative disorders (e.g., chronic lymphocytic leukemia). Lymphocytopenia may be due to infections (especially HIV), immunosuppressive therapy, medications (e.g., corticosteroids) and inherited immunodeficiency syndromes. Lymphocyte subsets (e.g., CD4, CD8) can be determined by flow cytometry.		Hematologic, CBC
Mean corpuscular hemoglobin (MCH), blood	H 4.2	26.5–34.0 pg^a^	MCH is a measure of the average quantity of hemoglobin (Hgb) per red blood cell (RBC). It is calculated by dividing hemoglobin by the RBC count (MCH = Hgb x 10/RBC count). MCH and mean corpuscular volume (MCV) are related such that when MCV is low, MCH is also low. When MCH is low, the oxygen-carrying capacity of the blood is reduced.	The most common cause of low MCH is iron deficiency. Elevated MCH may be seen in megaloblastic anemia due to folate or vitamin B_12_ deficiency.	^40^	CBC
Mean corpuscular hemoglobin concentration (MCHC), blood	H 4.2	Males: 31.5–36.3%^a^ Females: 31.4–36.0%^a^	MCHC is the average concentration of hemoglobin (Hgb) per red blood cell. It is calculated by dividing hemoglobin by hematocrit (Hct) (MCHC = Hgb x 10/Hct).	MCHC is increased in spherocytosis and homozygous hemoglobin C and sickle cell anemia. Since the MCHC is calculated from the Hgb and Hct, inaccuracies in their measurement (e.g., due to hyperlipidemia or hyperbilirubinemia), will result in artificially high MCHC. Free plasma hemoglobin due to hemolysis can also cause a falsely high MCHC. Though MCHC is decreased in iron deficiency anemia, it is not a sensitive test for this condition.	^40^	CBC
Mean corpuscular volume (MCV), blood	H 4.2	78.2–97.9 fL	MCV is directly measured by many automated hematology analyzers or can be calculated from hematocrit (Hct) and red blood cell (RBC) count (MCV = Hct x 10/red blood cells). When MCV is increased, RBCs are called macrocytic; when MCV is low, they are microcytic.	MCV is elevated in reticulocytosis (e.g., hemolytic anemia), megaloblastic anemia (e.g., vitamin B_12_ or folate deficiency) and myelodysplasia. MCV may be falsely elevated in red blood cell agglutination. MCV is decreased in a variety of anemias including iron deficiency anemia, anemia of chronic disease, thalassemia/thalassemia trait and sideroblastic anemia.	^40^	CBC
Mercury, blood	CHEM 1.7	<10 ng/mL	Mercury is primarily absorbed from the GI tract. It can bind to sulfhydryl groups on proteins. It is lipophilic, which enables it to cross the placenta and to concentrate in lipid-rich tissues such as the central nervous system. Because it is cleared by the kidney, renal damage may result.	Mercury affects the motor, sensory, cognitive and behavioral functions of the brain. Acute toxicity is associated with renal tubular injury and oliguria or anuria. Mercury exposure in utero can cause major CNS pathology, cerebral palsy and blindness. Vomiting and abdominal pain may develop upon acute ingestion.	^26^	Toxicology
Metanephrines (free), plasma or 24 h urine	CHEM 1.3	Plasma: <0.50 nmol/L. Urine: Normotensive adult males: 44–261 μg/24 h Normotensive adult females: 30–180 μg/24 h Hypertensive adults:< 400 μg/24 h	Metanephrines are two of the main metabolites of norepinephrine and epinephrine, two hormones that are secreted by pheochromocytomas and other tumors of neural crest origin. Measurement of metanephrines is more accurate than directly measuring epinephrine or norepinephrine. Symptoms associated with catecholamine-secreting tumors include hypertensive episodes, sweating, palpitations and headaches.	Measurement of plasma free metanephrines has very high sensitivity for pheochromocytoma and paraganglioma. Elevated metanephrine levels are suggestive of neural crest tumors but should be confirmed by a second test (24-hour urine metanephrines).		Neurologic
Monocyte count, blood	H 4.2	0.26–0.81 × 10^9^/L	Monocytes are a component of the innate immune system. They circulate in the blood before differentiating into macrophages and dendritic cells.	Monocyte numbers increase in chronic infection (e.g., tuberculosis), chronic inflammation (e.g., autoimmune disease) and myeloid neoplasms (e.g., chronic myeloid leukemia). Monocyte levels may be decreased in inherited immune disorders, corticosteroid therapy, chemotherapy, some infections and hairy cell leukemia.		CBC
N-type and P/Q-type calcium channel antibodies, serum	CHEM 1.6	N-type: ≤ 0.03 nmol/L P/Q type: ≤ 0.02 nmol/L	N-type or P/Q type voltage-gated calcium channels (VGCCs) are expressed on presynaptic axon terminals and regulate presynaptic neurotransmitter release. Antibodies against these proteins mediate cross-linking of channel subunits, reducing channel cell surface expression and thereby attenuating channel activity. Of the two anti-VGCC antibody subtypes, anti-P/Q antibodies are more common.	Anti-VGCC antibodies are seen in Lambert-Eaton myasthenic syndrome (LEMS), an autoimmune disease of presynaptic terminals; the majority of cases (50–60%) occur in association with small cell lung cancer. Impaired neurotransmitter release from the lower motor neurons, sympathetic neurons, and parasympathetic neurons in LEMS results in weakness, reduced tendon reflexes and autonomic dysfunction.	^49^	Nervous
Neutrophil count, blood	H 4.2	1.56–6.45 × 10^9^/L	Neutrophils are phagocytic white blood cells that are important in acute inflammation. They are the most plentiful WBC in the peripheral blood of adult patients. Mature neutrophils have segmented nuclei; immature neutrophils have unsegmented, S- or U- shaped nuclei (“band forms”). Neutrophils contain granules with antimicrobial compounds, such as myeloperoxidase, elastase and lysozyme. Neutrophils generate antimicrobial reactive oxygen species via a respiratory burst.	Absolute neutrophilia is seen in acute infections (especially bacterial and fungal), tissue necrosis, growth factor (granulocyte colony stimulating factor, G-CSF) effect, corticosteroid therapy and chronic myeloid neoplasms. “Left shift” refers to the presence of an increased proportion of band neutrophils and is characteristic of acute infection. Neutropenia is primarily due to destruction or decreased production of neutrophils. Causes include medications (e.g., chemotherapy), radiation, certain infections, autoimmune disease and bone marrow failure (e.g., myelodysplastic syndromes).		CBC
Nontreponemal syphilis tests (rapid plasma reagin [RPR], venereal disease research laboratory [VDRL], serum/cerebrospinal fluid (CSF)	M 2.13	Non-reactive	VDRL and RPR are non-treponemal serologic tests that measure antibodies to lipoprotein/cardiolipin antigens that are released from cells damaged by *Treponema pallidum*, the etiologic agent of syphilis. Titers decrease with time and following treatment, so these tests are most useful in the diagnosis of primary and secondary syphilis infection and in following response to therapy. Antibodies are detectable with both tests within a few weeks of development of the primary chancre, about 4–6 weeks after infection.	Titers between different non-treponemal tests and are not comparable, so the same test should be used to monitor response to treatment. Because the antibodies are not specific for syphilis, a treponemal test (e.g., *T. pallidum* IgG/IgM antibody test) is required for confirmation. Nontreponemal serologic tests use cardiolipin antigens in the analysis, which can lead to false positive in patients with systemic lupus erythematosus (SLE) who have antiphospholipid antibodies.		Genitourologic/Gynecologic
Oligoclonal bands, cerebrospinal fluid (CSF)	CHEM 1.8	<2 unique bands	IgG in the CSF is indicative of central nervous system (CNS) inflammation. Electrophoresis of the patient's CSF and serum normally yields no or at most one band. When two or more bands are seen in the CSF that are not in the serum, it is likely there is an autoimmune or infectious response within the CNS.	Oligoclonal bands are seen in the setting of CNS inflammation such as in multiple sclerosis (MS), CNS syphilis, progressive multifocal leukoencephalopathy and Guillain-Barre syndrome. Up to 95% of patients with MS are positive for oligoclonal bands, though additional clinical and imaging studies are necessary for diagnosis.		Central nervous
Oral glucose tolerarance test, blood	CHEM 1.3	Fasting: <95 mg/dL 1 h glucose level: < 180 mg/dL 2 h glucose level: < 155 mg/dL 3 h glucose level: <140 mg/dL	Glucose metabolism is impaired in diabetes, including gestational diabetes. For an oral glucose tolerance test, the patient ingests a standard amount of glucose and blood glucose levels are measured at defined time points. There are separate reference ranges for the various time points.	This testing is used most commonly in evaluation of gestational diabetes but can also play a role in the diagnosis of type 2 diabetes.		Endocrine
Parathyroid hormone, serum	CHEM 1.3	Adults: 15–65 pg/mL	PTH is synthesized and secreted by the chief cells of parathyroid glands. It plays a crucial role in maintaining calcium homeostasis by acting directly on bone and kidney, and indirectly on the intestines through 1,25-dihydroxyvitamin D. PTH promotes osteoclastic bone resorption and calcium and phosphate release. In the kidney, PTH stimulates calcium reabsorption and inhibition of phosphate reabsorption. These actions increase the plasma concentration of free calcium and decrease plasma phosphate concentration. The primary regulators of PTH secretion are calcium, 1,25-dihydroxyvitamin D, and phosphate.	Determination of PTH is useful in the differential diagnosis of both hypercalcemia and hypocalcemia. In hypercalcemia due to primary hyperparathyroidism (adenoma, hyperplasia), patients have increased PTH levels. In hypercalcemia due to other causes (e.g., PTHrP in malignancy), PTH is typically low. Secondary hyperparathyroidism is compensatory oversecretion of PTH due to abnormally low serum calcium (e.g., renal failure, gastrointestinal malabsorption, vitamin D deficiency). The most common causes of hypoparathyroidism are parathyroidectomy or thyroidectomy with accidental removal of parathyroids. The short half-life of PTH makes it possible to measure PTH levels intraoperatively during surgeries for primary hyperparathyroidism to assess effectiveness of resection of the parathyroids.	^50^	Endocrine
Parathyroid hormone- related peptide (PTHrP), plasma	CHEM 1.3	≤4.2 pmol/L	Parathyroid hormone related peptide (PTHrP) is secreted in low levels by most tissues and binds to the same receptor as parathyroid hormone. Elevation of PTHrP is often part of a paraneoplastic syndrome and causes humoral hypercalcemia of malignancy by stimulating calcium resorption in bone and calcium reabsorption in the kidney. Elevated PTHrP is most commonly produced by breast carcinoma, squamous cell carcinoma of lung and squamous cell carcinoma of the head and neck.	In humoral hypercalcemia of malignancy, parathyroid hormone levels are generally low or undetectable due to feedback inhibition. PTHrP can be elevated in benign conditions such as pregnancy and systemic lupus erythematosus (SLE) and others. Successful treatment of the underlying malignancy usually results in decreased levels of PTHrP and calcium and subsequent increases in parathyroid hormone levels.		Skeletal
Parietal cell antibodies, serum	IMM 1.4	Negative: ≤20.0 U Equivocal: 20.1–24.9 U Positive: ≥ 25.0 U	Parietal cell antibodies are IgG that bind to the H^+^/K^+^ ATPase pump on gastric parietal cells. They are seen in autoimmune gastritis, a T-cell mediated inflammatory condition that leads to loss of parietal cells, atrophy and metaplasia of the oxyntic mucosa, and, if chronic, pernicious anemia, caused by loss of intrinsic factor and decreased vitamin B_12_ absorption.	Parietal cell antibodies are found in more than 90% of patients with pernicious anemia, but are less specific than intrinsic factor antibodies since they may be positive in patients with other forms of chronic gastritis and in patients with thyroid disorders. False positives can occur when patients have received intramuscular or subcutaneous vitamin B_12_.		Gastrointestinal
Peripheral smear, blood	H4.3	Not applicable	A peripheral blood smear is made by spreading a drop of blood on a glass slide and staining the slide with Wright or Wright-Giemsa stain. This test is useful for investigating abnormal results from automated testing.	Review is used to evaluate red blood cell morphology (e.g., sickle cells, poikilocytosis) and atypical white blood cells (e.g., blasts), to confirm low platelet counts, and occasionally to identify microbial pathogens (e.g., malaria and babesia).		CBC
Phospholipase A2 receptor (PLA2R) antibody, serum	CHEM 1.5	Negative	Membranous nephropathy (MN) is a renal disease in which immune complexes deposit along the subepithelial surface of the glomerular basement membrane. In about 70% of cases of primary MN, the immune complexes are directed against the podocyte PLA2R protein.	The PLA2R antibody test can be used to differentiate primary from secondary MN and, with eGFR and proteinuria, correlates with risk of disease progression. PLA2R levels can also be used to monitor response to treatment.		Renal
Platelet count, blood	H 4.2	Males: 135–317 × 10^9^/L Females: 157–371 × 10^9^/L	Platelets are central to primary hemostasis. They interact with von Willebrand factor and exposed collagen to form a platelet plug at sites of endothelial injury. They also have receptors for fibrinogen, allowing for fibrin formation/crosslinking. A part of the platelet pool is normally sequestered in the spleen. Platelet counts may be falsely reported as low due to specimen clotting, platelet clumping and platelet satellitosis in which platelets adhere to neutrophils. Hematology analyzers count platelets based on size and do not recognize these aggregates as platelets.	Causes of thrombocytopenia are sequestration (e.g., hypersplenism), increased consumption (e.g., heparin-induced thrombocytopenia, immune thrombocytopenic purpura, disseminated intravascular coagulation, thrombotic thrombocytopenic purpura) or decreased production (infiltration of bone marrow, leukemias, viral infections). Causes of thrombocytosis include inflammation, hyposplenism/splenectomy, iron deficiency and myeloproliferative neoplasms. Even if counts are normal, platelets may be dysfunctional due to drugs (e.g., aspirin), uremia and genetic diseases (e.g., Bernard-Soulier syndrome, Glanzmann thrombasthenia).		CBC
Porphobilinogen and aminolevulinic acid, plasma	H 4.4	Porphobilinogen: ≤ 0.5 nmol/mL Aminolevulinic Acid: ≤ 0.5 nmol/mL	The porphyrias are a group of diseases caused by abnormalities in the enzymes involved in heme synthesis. Intermediates accumulate or are deficient, depending on the enzyme affected. The first step of the heme biosynthetic pathway involves synthesis of aminolevulinic acid (ALA) from succinyl CoA and glycine through the action of ALA synthase. ALA dehydratase, the next enzyme in the pathway, converts ALA to porphobilinogen (PBG).	The porphyrias are subclassified based on their clinical presentation (acute versus cutaneous). Acute porphyrias can present with attacks of neuropsychiatric symptoms, discolored urine and abdominal pain. Cutaneous porphyrias are associated with skin manifestations and photosensitivity related to accumulation of porphyrins in the skin. Serum ALA and porphobilinogen testing are part of the initial evaluation of porphyria. Both are elevated during acute attacks of acute intermittent porphyria, hereditary coproporphyria and variegate porphyria. Elevation of ALA without PBG can be seen in aminolevulinic acid dehydratase deficiency porphyria, tyrosinemia and heavy metal exposure. ALA dehydratase is inhibited by lead.		Hematologic
Potassium, serum	CHEM 1.2 and CHEM 1.4	3.6–5.2 mmol/L	Potassium, K^+^, is the main intracellular cation, while sodium is the main extracellular cation. The Na^+^/K^+^ ATPase membrane pump maintains the concentrations of these 2 cations in their respective compartments. Plasma level is regulated by the kidney. Abnormally high or low potassium concentrations interfere with muscle contraction including the myocardium, and nerve conduction; low levels increase the cell membrane potential while high levels decrease the membrane potential.	Potassium is part of a basic metabolic panel. Both hypo- and hyperkalemia can lead to cardiac arrhythmias. Important causes of hypokalemia include medications (e.g., diuretics), vomiting, diarrhea and diabetic ketoacidosis. Hyperkalemia may be due to medications (e.g., angiotensin coverting enzyme [ACE] inhibitors), Addison disease, renal failure (decreased excretion) and extracellular potassium shift (e.g., secondary to diabetic ketoacidosis). Extensive cellular destruction (e.g., trauma, burns, hemolysis) can also lead to hyperkalemia. Potassium may appear falsely elevated in hemolyzed blood samples.	^23^	BMP/Chem 7
Progesterone, serum	CHEM 1.3	Adult females (non-pregnant): Follicular phase: ≤ 0.89 ng/mL Ovulation: ≤12 ng/mL Luteal phase: 1.8–24 ng/mL Pregnancy: 1st trimester: 11–44 ng/mL 2nd trimester: 25–83 ng/mL 3rd trimester: 58–214 ng/mL	Progesterone is produced by the corpus luteum, placenta, and the adrenal cortex. During the luteal phase of the menstrual cycle, progesterone helps prepare the endometrium for embryo implantation by promoting endometrial gland secretions and the development of spiral arteries.	Measurement of a midluteal progesterone level can be used to determine whether ovulation has occurred. In the absence of fertilization, the corpus luteum regresses, which causes progesterone levels to drop, and menses will occur as the uterine lining sheds.		Endocrine
Prolactin, serum	CHEM 1.3	Adult males: 4.0–15.2 ng/mL Adult females: 4.8–23.3 ng/mL	Prolactin is produced by lactotrophs of the anterior pituitary gland and is responsible for lactation. Its secretion is circadian and pulsatile with highest levels during sleep and is under the inhibitory control of dopamine produced by the hypothalamus. Prolactin levels rise during pregnancy and are highest in the third trimester; in the absence of breastfeeding, they return to baseline about three weeks after delivery. Prolactinomas are the most common adenomas of the pituitary (40% of total).	Common causes of elevated prolactin levels are prolactinoma, diseases of the hypothalamus, primary hypothyroidism and polycystic ovary syndrome. Hyperprolactinemia may cause galactorrhea (nipple discharge), menstrual cycle disruption, infertility, and loss of libido.		Endocrine
Prostate specific antigen (PSA), serum	CHEM 1.8	Total PSA 0–4 ng/mL	PSA is a protease secreted by the epithelial cells of the acini and ducts of the prostate gland that is found in the blood in protein-bound and free forms.	Total PSA (bound + unbound) is a marker for prostate cancer and is useful in diagnosis, staging and monitoring treatment, though it is not specific for malignancy. PSA elevations can also be seen in prostatitis, benign prostatic hyperplasia and after procedures and ejaculation. Definitive diagnosis of prostate cancer requires biopsy and pathologist examination. Higher rates of change in PSA levels (> 0.75 μg/L/yr) are seen in patients with prostate cancer.		Urologic
Protein C activity, plasma	H 2.4	70–150%	Protein C is a vitamin K-dependent anticoagulant synthesized by hepatocytes. Binding of thrombin to the endothelial cell receptor thrombomodulin activates protein C. Activated protein C (APC), combined with protein S, inactivates factors Va and VIIIa, which are needed for thrombin generation.	Protein C deficiency is a rare cause of inherited thrombophilia and may also be secondary to vitamin K deficiency, severe liver disease, medications and disseminated intravascular coagulation. Protein C deficiency can lead to skin necrosis when warfarin treatment is initiated and neonatal purpura fulminans in neonates.		Coagulation
Protein S activity, plasma	H 2.4	Males: 65–160% Females <50 years: 50–160% ≥ 50 years: 65–160%	Protein S is a vitamin K-dependent anticoagulant synthesized by hepatocytes. It enhances the effect of activated protein C to accelerate the degradation of factors Va and VIIIa.	Protein S deficiency is a rare cause of inherited thrombophilia and may also be secondary to vitamin K deficiency, severe liver disease, medications and the nephrotic syndrome. Like protein C, protein S deficiency can lead to skin necrosis when warfarin treatment is initiated and, less commonly than protein C deficiency, can cause neonatal purpura fulminans in neonates.		Coagulation
Protein (total), serum	CHEM 1.4	6–8.5 g/dL	Serum total protein measures all circulating protein, which is primarily albumin (made in the liver) and globulins. Protein electrophoresis can be performed to differentiate the different contributions of these proteins.	Since total protein consists primarily of albumin, it is most informative to combine this test with an albumin level. When albumin is low and total protein is high, protein electrophoresis can identify which proteins are elevated.		Hepatic
Prothrombin G20210A mutation, blood	H 2.7	Negative	The prothrombin G20210A mutation confers an increased risk of venous thromboembolism (VTE). It is the second most common thrombophilic mutation (factor V Leiden is more common). The mutation is in the 3′ untranslated region and results in increased prothrombin function, perhaps due to increased prothrombin synthesis or stability.	The primary effect of this mutation is increased risk of VTE and a slight increase in risk of arterial thromboembolism. Heterozygote individuals have a 3- to 4-fold risk of VTE over baseline; due to the rarity of homozygote individuals, absolute risk cannot be calculated. This test is typically part of a panel in patients who are thrombophilic and includes factor V Leiden mutation, activated protein C resistance, antithrombin activity, protein C activity and protein S. Compound heterozygotes of factor V Leiden and the prothrombin G20210A mutation can be seen.	^51^	Coagulation
Prothrombin time (PT), plasma	H 2.4	PT: 9.4–12.5 s International normalized ratio (INR): 0.9–1.1	PT assesses the extrinsic pathway of the coagulation cascade. The test is performed by adding phospholipids, tissue factor, and calcium to the patient's plasma and measuring time to clot. Since tissue factor activity may vary in different batches and from different sources, PT results are typically standardized by converting the value into an International Normalized Ratio (INR) (see INR entry).	Prothrombin time is elevated in patients with a deficiency in or inhibition of factors VII, X, II (thrombin), or I (fibrinogen) due to a genetic deficiency or as a secondary effect (e.g., liver failure, warfarin therapy). A prolonged PT should be interpreted in the context of a concurrent PTT to determine whether the deficiency is in the extrinsic pathway or in the common pathway. A mixing study may be performed to distinguish a factor deficiency from inhibition. PT/INR is commonly used as a screening test or to monitor patients on warfarin therapy.		Coagulation
Quadruple marker test: Alpha-fetoprotein (AFP), unconjugated estriol (uE3), human chorionic gonadotropin (hCG), dimeric inhibin A (DIA), maternal serum	FDP 1.3	Down Syndrome: Calculated screen risks <1/270: screen negative Calculated screen risks ≥1/270: screen positive Trisomy 18: Calculated screen risks <1/100: screen negative Calculated screen risks ≥1/100: screen positive Neural Tube Defects: AFP multiple of the median (MoM) < 2.5: screen negative. AFP multiple of the median (MoM) < 2.5: screen negative. AFP MoMs ≥2.5: screen positive	The quadruple marker test, or quad screen, is a 2nd trimester prenatal screening test, which uses the levels of four biomarkers produced during pregnancy by the mother, the placenta, or the fetus (AFP, uE3, hCG, DIA) to estimate the risk of fetal chromosomal abnormalities, including trisomy 18 and 21, as well as neural tube and abdominal wall defects.	The Quad test is a screening test. Negative tests do not exclude an abnormality. Positive tests may indicate the need for further testing. Since the substrate is maternal blood, there is no risk to the fetus. Several factors may affect results including calculated gestational age, diabetes, multiple gestations (i.e., twins, triplets), maternal weight and maternal smoking.		Gynecologic
Rapid plasma reagin (RPR), serum	M 2.13	Non-reactive	The RPR test is a non-treponemal serologic test for syphilis that measures antibodies to lipoprotein/cardiolipin antigens that are released from cells damaged by *T. pallidum*; these antibodies are not specific for syphilis. RPR titers decrease with time; therefore this test is less sensitive for detection of tertiary and latent syphilis than for detection of primary and secondary syphilis. Antibodies are detectable with RPR within 1–4 weeks of development of the primary chancre.	RPR is often used for syphilis screening due to its low cost and ease of use; however both false positives and false negatives are possible. A treponemal antibody test should be used for confirmation or in the setting of a negative test if suspicion is high. RPR is semiquantitative and can be used to monitor therapy since titer decreases with successful treatment. Titers between different non-treponemal tests cannot be compared; therefore, the patient must be monitored with the same test over time. Nontreponemal serologic tests use cardiolipin antigens in the analysis, which can lead to false positive in patients with systemic lupus erythematosus (SLE) who have antiphospholipid antibodies.		Genitourinary/Gynecologic
Red blood cell (RBC) count, blood	H 4.2	Male: 4.35–5.65 × 10^12^/L Female: 3.92–5.13 × 10^12^/L	Red blood cell count is reported as the number of RBCs per L of blood, but gives no information about the size or functional status of the RBCs. RBC production is stimulated by erythropoietin (EPO), which is produced by the kidneys.	The RBC count is increased in polycythemia and decreased in anemia. In thalassemia trait, the RBC count is often higher than expected for the degree of anemia. Polycythemia is due to increased production (e.g., polycythemia vera, EPO administration, EPO-producing tumors), whereas anemia may be due to decreased production (e.g., iron deficiency, infiltrative marrow processes) or increased destruction (e.g., sickle cell anemia, hereditary spherocytosis). Inaccurate RBC counts may be due to agglutination. Microcytic RBCs and RBC fragments (e.g., schistocytes) may be erroneously counted as platelets by the automated analyzer.	^40^	CBC
Red blood cell distribution width (RDW), blood	H 4.2	Males: 11.8–14.5% Females: 12.2–16.1%	RDW is a measure of the variability in red blood cell (RBC) size. Increased RDW is seen when there is anisocytosis or when there is a dimorphic RBC population (i.e., two populations of differing size as is the case in recent transfusion in a patient with a microcytic anemia). The presence of reticulocytes also increases the RDW.	In the setting of microcytic anemia, increased RDW suggests iron deficiency anemia rather than thalassemia trait, which typically has more uniform cell sizes. An increased RDW in the setting of macrocytosis is more suggestive of vitamin B_12_ or folic acid deficiency than other causes of macrocytosis. Sideroblastic anemia is another cause of dimorphic RBC populations.	^40^	CBC
Renin activity, plasma	CHEM 1.5	Adults, normal sodium diet: 0.6–4.3 ng/mL/hr	The renal juxtaglomerular apparatus produces renin, an enzyme that converts angiotensinogen to angiotensin I, which is then further converted to angiotensin II. Angiotensin II stimulates the zona glomerulosa of the adrenal cortex to release aldosterone, which increases sodium reabsorption. Consequent water reabsorption causes a rise in systemic (and therefore renal) blood pressure. Renin secretion by the kidney is stimulated by a fall in glomerular blood pressure, by decreased sodium concentration at the macula and by stimulation of sympathetic outflow to the kidney.	Plasma renin activity (PRA) is measured as part of the diagnosis and treatment of hypertension. Interpretation is dependent on salt intake, posture, time of day, and certain medications. Increased PRA may indicate Addison disease, cirrhosis, essential hypertension, hemorrhage, hypokalemia, renin-producing renal tumors, and renovascular hypertension. Decreased PRA may can be seen in primary hyperaldosteronism, salt-retaining steroid therapy, and salt-sensitive essential hypertension. Renal disease, especially unilateral renal artery stenosis, results in elevated aldosterone and renin. A high ratio of serum aldosterone to renin activity suggests primary aldosteronism.		Renal
Reticulocyte count, blood	H 4.2	0.60–2.71%	Reticulocytes are immature red blood cells (RBCs) released from the bone marrow that are anucleate but still contain some organelles, such as ribosomes. They are slightly larger and more basophilic than mature RBCs due to retained RNA. The reticulocyte count reports reticulocytes as a percentage of total number of RBCs.	Reticulocyte count reflects recent bone marrow erythropoetic function. Elevated reticulocyte count is a normal physiologic response to anemia of any cause; the reticulocyte index may be calculated in a patient with anemia to determine if the bone marrow is responding appropriately to compensate for the anemia. However, in these patients, the reticulocyte count may be falsely elevated since it is reported as a percentage of RBCs (which are low in anemia).		CBC
Rh(D) type, serum	TM1.1, TM1.6	Positive or negative, varies by population	The Rh system demonstrates >50 antigens, the most important of which is the D antigen. Antibody to D antigen (anti-D) is acquired after Rh(D)negative individuals are exposed to the D antigen (transfusion, pregnancy, transplant).	Women who are Rh(D) negative and have a fetus who is Rh(D) positive can have a healthy pregnancy; however, in subsequent pregnancies, an Rh(D) positive fetus is at risk of hemolytic disease of the fetus and newborn (HDFN). Therefore, Rh(D) negative women who are carrying an Rh(D) positive fetus are treated prophylactically with Rh(D) immunoglobulin at 28 weeks' gestation and within 72 hours of delivery. Anti-D can also cause a mild to severe immediate or delayed hemolytic transfusion reaction.	^52^	Transfusion
Rheumatoid factor (RF), serum	IMM 1.4	<15 IU/mL	RF is an antibody that reacts with the Fc portion of other immunoglobulin G antibodies. Despite its name, RF lacks specificity for rheumatoid arthritis (RA) and can be seen in other inflammatory conditions and autoimmune diseases, particularly Sjögren syndrome.	RF can be a prognostic indicator in RA as its presence correlates with increased severity of disease. RF has a sensitivity and specificity of about 70% and 85% for RA, respectively. Combination of RF and anti-citrullinated peptide antibodies have a higher diagnostic yield than RF alone.		Skeletal, Autoimmunity
Salicylate, serum	CHEM 1.7	Therapeutic: < 30 mg/dL	Aspirin has a short half-life (15 min) and is rapidly metabolized to salicylate. In aspirin overdose, cyclooxygenase inhibition leads to platelet dysfunction and gastric mucosal injury; stimulation of the medulla resulting in hyperventilation, respiratory alkalosis, nausea and vomiting; and disruption of cellular metabolism (e.g., oxidative phosphorylation) which can cause metabolic acidosis.	Serum salicylate levels above 40 mg/dL typically result in toxicity. Symptom onset is usually within 3–8 h, but absorption rates are not uniform. Timing and concentration of peak serum levels can vary depending on the amount and form of aspirin ingested; therefore, serial measurements every two hours are recommended until two consecutive tests show decreasing concentration. Acetaminophen levels should also be ordered since some formulations have both compounds.		Toxicology
Sodium, serum	CHEM 1.5 and CHEM 1.3	135–145 mmol/L	Sodium, Na^+^, is the main extracellular cation while potassium, K^+^, is the main intracellular cation. The Na^+^/K^+^ ATPase membrane pump maintains the concentrations of these two cations in their respective locations. When extracellular Na^+^ levels decrease, water shifts into cells and vice versa. With chronically low levels of Na^+^, cells adapt and patients may be asymptomatic, but acute hyponatremia can result in seizures or brain herniation due to cerebral edema. There are two main hormones that are important in Na^+^ regulation: arginine vasopressin (antidiuretic hormone [ADH]) and aldosterone. Abnormal Na^+^ concentrations are usually due to renal dysfunction or changes in intake.	Na^+^ is the electrolyte that is most commonly abnormal. Hyponatremia is usually due to excess hydration, not insufficient intake, and can be seen in renal failure, primary polydipsia, thiazide diuretics, SIADH, and adrenal insufficiency. Rapid correction of hyponatremia can lead to osmotic demyelination syndrome. Hypernatremia is often due to fluid depletion (e.g., vomiting, diarrhea, dehydration, osmotic diuresis in uncontrolled diabetes) though it can also be due to excess intake (e.g., saline emetic, hypertonic sodium bicarbonate to treat metabolic acidosis). Symptoms include signs of dehydration such as thirst, headache, lethargy, and weakness. When untreated, severe hypernatremia can cause spasms, seizure, and coma.		BMP/Chem 7
T3 (triiodothyronine), total, serum	CHEM 1.1	80–200 ng/dL	T3 (triiodothyronine) is the most physiologically active form of thyroid hormone. Only a small percentage of thyroid hormone is released as T3; the rest is thyroxine or T4. T4 is slowly deiodinated to T3 once in circulation. Several tests may be used to evaluate T3 including total T3 and free T3 (measures unbound T3).	T3 levels are assessed in conjunction with thyroid stimulating hormone (TSH), total and free T4 to evaluate thyroid function and to assess treatment for thyroid disease. T3 and free T3 are not routinely used for this purpose, since total and free T4 are sufficient in most cases. T3 is used to evaluate thyrotoxicosis.	^53^	Endocrine
T4 (thyroxine), free, serum	CHEM 1.1	Adults: 0.9–1.7 ng/dL	Thyroxine (T4) is synthesized in the thyroid gland and metabolized peripherally to triiodothyronine (T3). The majority of T4 is bound to thyroid binding globulin; free T4 is the active form. Measurement of total T4 was once routine for thyroid assessment; however, interpretation is complicated by variation in binding proteins across individuals and physiologic conditions. Although only about 0.05% of circulating T4 is unbound to binding proteins (“free”), measurement of free T4 provides an accurate assessment of thyroid status in most patient populations. Total T4, free T4 and thyroid stimulating hormone (TSH) are often used together to evaluate thyroid function.	Low free T4 is seen in hypothyroidism; high free T4 is seen in hyperthyroidism. Free T4 should be assessed in conjunction with TSH, the pituitary hormone that regulates thyroid hormone production. A common algorithm is TSH measurement, which reflexes to free T4 if TSH concentration is abnormal.		Endocrine
Testosterone, serum	CHEM 1.3	Varies with age and sex	Testosterone is the main endogenous androgen. In males, testosterone controls development of external genitalia and secondary sexual characteristics. The testicular Leydig cells are the main source of testosterone, with the adrenal cortex producing lesser amounts. In females, testosterone is primarily a precursor to estrogens, with the ovaries as the main source of production. Testosterone production in testes and ovaries is regulated via pituitary-gonadal feedback involving luteinizing hormone (LH) and, to a lesser degree, inhibins and activins.	Excess testosterone can manifest as precocious puberty in boys, masculinization in girls and virilization in women. Polycystic ovarian syndrome is a relatively common condition characterized by elevated testosterone in females. Elevated testosterone may also be due to testicular or adrenal tumors, anabolic steroid use and congenital adrenal hyperplasia (in babies and children). Testosterone levels may be assessed in adult males and females with reduced libido.		Endocrine
Thyroid-stimulating hormone (TSH), serum	CHEM 1.1	Adults: 0.3–4.2 mIU/L	TSH (thyrotropin; thyroid stimulating hormone) is produced by the anterior pituitary with feedback inhibition by thyroid hormones. It interacts with cell receptors on the thyroid follicular cells to stimulate cell division, cell hypertrophy and increased synthesis of thyroid hormones (thyroxine and triiodothyronine).	TSH is the primary screen for primary hypothyroidism and primary hyperthyroidism. When TSH is low, free T4 and T3 are added to determine the extent and etiology of thyroid dysfunction. When TSH is high, free T4 is performed to assess the degree of hypothyroidism. For patients with primary hypothyroidism who are treated with levothyroxine, TSH alone is a sufficient monitoring test. For patients with central hypothyroidism (hypothyroidism caused by hypothalamic or pituitary disease), free T4 is low or low-normal and the TSH may be low or normal.	^53^^,^^54^	Endocrine
Thyroid peroxidase antibody, serum	CHEM 1.1	<9.0 IU/mL	Thyroid peroxidase (TPO) catalyzes iodination of thyroglobulin to form monoiodotyrosine and diiodotyrosine, precursors of thyroid hormone. Antibodies against TPO are common in autoimmune thyroid disease, though they are also present in 5–20% of the general population. They are polyclonal, which suggests they are secondary phenomena to thyroid injury. Anti-TPO antibodies can activate complement and likely play a key role in the pathogenesis of autoimmune-mediated hypothyroidism.	Anti-TPO antibodies are sensitive tests for autoimmune thyroid disease (e.g., Hashimoto thyroiditis, Graves disease) but are not specific within this class of disorders. The highest anti-TPO levels are generally seen with Hashimoto thyroiditis.	^53^	Endocrine, Autoimmunity
Thyrotropin (thyroid stimulating hormone, TSH) receptor antibody, serum	CHEM 1.1	≤1.75 IU/L	In Graves disease, TSH receptor antibodies (also called thyroid-stimulating immunoglobulin) bind to and activate the TSH receptor without feedback inhibition, causing thyrotoxicosis. TSH receptor antibodies that block the receptor may also be seen and it is thought that the balance between blocking vs activating antibodies may contribute to disease severity in Graves disease.	TSH receptor antibody testing is useful when Graves disease is suspected clinically, but thyroid function tests are normal and in patients for whom radioisotope testing is contraindicated (e.g., during pregnancy). TSH receptor antibodies may persist even with successful surgical or ablation therapy; since these are IgG antibodies, they can cross the placenta and cause neonatal thyrotoxicosis.		Endocrine, Autoimmunity
Tissue transglutaminase antibody, IgA, serum	IMM 1.4	Negative: <4.0 U/mL Weak positive: 4.0–10.0 U/mL Positive > 10.0 U/mL	Tissue transglutaminase (tTG) deamidates gliadin, which is bound with increased affinity by HLA-DQ2 and DQ8 molecules on antigen presenting cells, leading to a CD4^+^ T cell response. Injured or inflamed endothelial cells and fibroblasts release tTG. tTG autoantibodies are elevated in patients with celiac disease; therefore, this is the first-line screening test, in conjunction with biopsy, to confirm the diagnosis.	The test queries IgA tTG antibodies and will be negative in patients with IgA deficiency (about 2% of patients with celiac disease). Patients who have been on a gluten-free diet may have a negative result and the test is therefore useful in monitoring adherence to a gluten-free diet in patients with celiac disease. When combined with an endomysial antibody test, it is useful for screening for dermatitis herpetiformis.	^33^	Gastrointestinal, Autoimmunity
ToRCH Antibody, serum	M 3.3	*Toxoplasmosis gondii* antibody: Negative Rubella antibody: Vaccinated, Positive; Unvaccinated, Negative Cytomegalovirus (CMV) antibody: Negative Herpes simplex Virus (HSV) type 1 and 2 antibodies: Negative	Perinatal infections are responsible for 2–3% of congenital anomalies. *Toxoplasma gondii*, CMV, Rubella and HSV type 1/2 are assessed in pregnancy since while the patient may be asymptomatic, the fetus can be severely affected due to transplacental spread. “Other” entities (e.g., syphilis, human immunodeficiency virus, parvovirus B19) may be tested for separately. Positive results to the IgG test indicate past exposure or immunization. A positive result for IgM antibodies indicates current or recent infection.	In utero ToRCH infections are associated with growth retardation, fetal demise, hepatosplenomegaly, jaundice and lethargy. Additional findings such as distinctive rashes or chorioretinitis may suggest a specific diagnosis.		Gynecologic
Total cholesterol, serum	CHEM 1.2	Desirable: <200 mg/dL Borderline high: 200–239 mg/dL High risk: ≥ 240 mg/dL	Total cholesterol includes high-density lipoprotein (20–30%), low-density lipoprotein (60–70%) and very-low-density lipoprotein (10–15%). Low-density lipoprotein (LDL) is usually calculated from total cholesterol, high-density lipoprotein (HDL) and triglycerides. About 75% of the total cholesterol is synthesized by the liver and 25% comes from the diet.	Total cholesterol is elevated in a number of conditions including familial hypercholesterolemia (deficiency of LDL receptors), uncontrolled diabetes, hypothyroidism, nephrotic syndrome and biliary obstruction. Corticosteroids also increase total cholesterol. Total cholesterol may be decreased in severe liver disease, hyperthyroidism, severe acute or chronic illness, malnutrition, malabsorption and extensive burns.		Vascular
Total iron binding capacity (TIBC), serum	H 4.1 and CHEM 1.7	250–400 μg/dL	In the serum, iron is bound to transferrin, which is typically about one third saturated with iron. When iron stores in the body are depleted (e.g., iron deficiency anemia), transferrin levels increase in the blood, which increases the total iron binding capacity (TIBC).	In iron deficiency, TIBC is increased, since transferrin levels are relatively high compared to iron content; in iron overload, TIBC decreases since the free transferrin diminishes. TIBC, serum iron and percent saturation are often assessed in the setting of iron deficiency anemia; however, serum ferritin is more sensitive and more accurately reflects the body's iron stores.		Hematologic
*Treponema pallidum* IgG/IgM antibody, serum	M 2.13 and M 1.3	Non-reactive	A test for IgG and IgM antibodies to *T. pallidum* is a specific test for the causative agent of syphilis. This test is particularly useful in diagnosing tertiary and latent syphilis. However, these antibodies persist after infection and can be seen in treated individuals. Therefore, a second method such as the non-treponemal RPR or VDRL tests can help establish how recent the infection is and whether or not the patient has received adequate treatment.	This test is particularly useful in diagnosing tertiary and latent syphilis.		Genitourinary/Gynecologic
Triglycerides, serum	CHEM 1.2	Normal: <150 mg/dL Borderline high: 150–199 mg/dL High: 200–499 mg/dL Very high: ≥ 500 mg/dL	Triglycerides (TGs), low-density lipoprotein (LDL) and high-density lipoprotein (HDL) are the primary lipids found in plasma. Triglycerides are transported from the small bowel inside chylomicrons and as very low-density lipoprotein (VLDL) particles. TGs are directly measured in the laboratory. This value, along with total cholesterol and HDL, is used to calculate LDL. Patients with extremely elevated TGs may have inaccurate LDL results.	Elevated triglycerides are an independent risk factor for coronary artery disease as well as a risk factor for acute pancreatitis. Triglycerides are increased in wide range of conditions including diabetes, nephrotic syndrome, biliary tract obstruction, obesity, cirrhosis and some glycogen storage diseases. Many medications increase triglycerides: β-blockers, cholestyramine, corticosteroids, diazepam, diuretics and estrogens.		Vascular
Troponin T, high sensitivity (hs-cTnT), plasma	CHEM 1.2	Males: ≤ 20 ng/L Females: ≤ 15 ng/L	Troponin is a regulatory protein of striated muscle composed of three subunits: T, C, and I. The T, or tropomyosin-binding subunit, binds to muscle fibers. cTnT is specific for cardiac muscle and is released in myocardial cell death, about 2–3 hours after acute myocardial infarction. Because cTnT is bound to muscle fibers, it is released slowly into the peripheral blood following myocardial infarction. Its concentration peaks at 24 hours but it can persist 2 weeks or longer in the blood.	Hs-cTnT assays are preferred over conventional tests, which are relatively insensitive; for example, the former are reported as ng/L while the latter are reported as ng/mL. With hs-cTnT tests, low levels of troponin are detectable in healthy individuals. In addition to myocardial infarction, elevated cTnT can be seen in cardiac contusion, congestive heart failure, renal failure, pulmonary embolism and myocarditis. Biotin (vitamin B_7_), which is found in many multivitamins, interferes with the assay and can cause inaccurate results.	^55^^,^^56^	Cardiac
Uric acid, serum	CHEM 1.5	Males: < 8.0 mg/dL Females: < 6.1 mg/dL	Uric acid is generated by purine metabolism. Purines are synthesized by the body or are ingested, particularly in foods with abundant nucleic material (e.g., liver). About 75% of the body's uric acid is excreted in the urine.	Hyperuricemia can be seen in patients on cytotoxic drug regimens (e.g., cancer chemotherapy) and in the context of gout, leukemia, chronic renal failure (decreased excretion) and psoriasis. Most patients with hyperuricemia do not develop gout.		Skeletal, Renal
Venereal disease research laboratory (VDRL) test, spinal fluid	M 2.13	Non-reactive	The VDRL test is a non-treponemal serologic test for syphilis that measures antibodies to lipoprotein/cardiolipin antigens that are released from cells damaged by *T. pallidum*; these antibodies are not specific for syphilis.	The VDRL test is often used for syphilis screening due to its low cost and ease of use; however both false positives and false negatives are possible. A treponemal test (such as an antibody test) should be used for confirmation or in the setting of a negative test if suspicion is high. A spinal fluid sample that is positive by the VDRL test is very specific for neurosyphilis; however, false negatives are common. Non-treponemal serologic tests use cardiolipin antigens in the analysis, which can lead to false positives in patients with systemic lupus erythematosus (SLE) who have antiphospholipid antibodies.		Genitourinary/Gynecologic
Vitamin B_12_ (cobalamin), serum	H 3.2	180–914 ng/L	Vitamin B_12_ (cobalamin) is a water-soluble vitamin. Uptake requires binding to intrinsic factor (IF) produced by parietal cells in the stomach and absorption in the terminal ileum. Vitamin B_12_ is required for the conversion of homocysteine to methionine in a process that yields tetrahydrofolic acid (TH_4_), which is required for the synthesis of deoxythymidine monophosphate (dTMP), a building block for DNA.	The most common cause of vitamin B_12_ deficiency is chronic atrophic gastritis (pernicious anemia), an autoimmune disease in which autoantibodies attack gastric parietal cells and IF. Vitamin B_12_ deficiency leads to megaloblastic anemia and subacute combined degeneration of the spinal cord. Subacute combined degeneration is characterized by degeneration of the dorsal and lateral columns of the spinal cord due to demyelination. It commonly presents with sensory deficits, paresthesia, weakness, ataxia, and gait disturbances. Neurologic symptoms may occur without any discernible hematologic changes in the blood.		Hematologic
Von Willebrand factor (vWF) antigen, plasma	H 2.1	55–200%	vWF is synthesized by endothelial cells and megakaryocytes. vWF participates in primary hemostasis by binding to platelet receptor GPIb-IX and by binding to subendothelial collagen. vWF binds and stabilizes factor VIII in circulation. The vWF antigen test measures the quantity of vWF.	This test is generally used in combination with a test evaluating the function/activity of vWF such as the vWF:ristocetin cofactor assay. Decreased levels of vWF can be seen in association with inherited or acquired forms of von Willebrand disease. The ratio of vWF antigen to activity level can help determine if there is a deficiency in production or in function of vWF.		Coagulation
White blood cell (WBC) count, blood	H 4.2	3.4–9.6 × 10^9^/L	WBCs are counted using an automatic analyzer. The WBC count may or may not include a differential count (a separate count of each type of WBC). Most labs perform an automated differential count as part of the WBC count. When abnormal cells are detected, a manual review of the blood smear is performed.	Increased WBCs are most often due to infection (i.e., neutrophils) or hematologic malignancies (often lymphocytes or myeloid cells). A low WBC count is usually a reflection of impaired marrow production due to medication, hematologic or metastatic malignancies or marrow infiltration (e.g., fibrosis, granulomas). In severe sepsis, there may be a paradoxical drop in the WBC count due to consumption in “neutrophil extracellular traps” (NETs).		CBC

a Duke University Hospital System (DUHS) reference value.

As laboratory tests have varying, population-dependent reference ranges, a uniform reference range that could be used for all included tests was sought. The Mayo Clinic graciously permitted the use of their normal reference ranges for all laboratory tests within ELTME. Common references cited for creation of the table include the Test Catalog from Mayo Clinic Laboratories,^5^ *Henry's Clinical Diagnosis and Management by Laboratory Methods*,^6^ *Tietz Textbook of Clinical Chemistry and Molecular Diagnostics*^7^*, Guide to Diagnostic Tests*^8^*, Robbins and Cotran Pathologic Basis of Disease*^9^*, and Blood Cells: A Practical Guide*.^10^ Specific additional references are noted in the table as appropriate.

## Discussion

As expected for the scope of this project, the development of the ELTME was a significant undertaking. On one level, this project addresses the essential pathophysiology of common laboratory tests. It was a challenging process to determine which tests would be most relevant to undergraduate medical education. Many laboratory tests, including those in specific areas such as microbiology and genomics, were briefly or not at all covered in an effort to keep the ELTME concise and easy to use.

We anticipate this reference will be a useful resource for medical educators and will facilitate incorporation of laboratory testing into curricula. With the increased adoption of integrated medical curricula, teaching time is often limited, and thoughtful, focused educational materials are essential. Many existing textbooks or references contain more information than a typical medical student will need to know, but due to clinical and administrative duties, medical educators may find it challenging to independently develop curricular materials such as this one. The easy searchability of this table enables educators to quickly identify the tests relevant to the material they are teaching and integrate them into teaching materials as appropriate. With simple editing tools, new tables may be constructed to fit the needs of individual curricula. Furthermore, the table may be easily expanded by educators who wish to add specific tests to complement their curricula.

The second intended audience for this publication is medical students, residents, and fellows who must master an enormous amount of material in a short time. This peer-reviewed, concise document of common laboratory tests addresses what each test measures and the associated pathophysiology. This combination will help provide a more thorough understanding of the basic science underlying disease and laboratory testing. By focusing on the pathophysiology of each laboratory test, the context for educated decision making is provided, thereby contributing to value-based patient care.

The PCME provided a framework for this project on laboratory testing. Medical educators have been using and adapting the PCME for their own curricula since its publication in 2017 and educational cases have been published to highlight the pathology for the PCME learning objectives. By combining the learning objectives for the PCME with corresponding laboratory tests in the ELTME, medical educators and learners can easily query the Association of Pathology Chairs (APC) website (https://www.apcprods.org/journal) to identify associated educational cases for curricular use or self-directed learning.

## Conclusion

Physician understanding of common laboratory tests is essential for effective clinical reasoning, diagnosis, and management. The ELTME consists of common laboratory tests essential to basic medical practice and highlights the pathophysiology and clinical pearls for each of those common laboratory tests, facilitating learner comprehension and providing further context. This document is intended to help medical educators, medical students, and resident and fellow physicians understand common laboratory tests, acquire clinical insights, and reference relevant educational cases that illustrate the importance of these tests as well as easily look at the pathology competency and identify educational cases that will further explain or give examples of use of laboratory tests.

## Authors’ note

The opinions expressed are those of the authors and do not reflect the official positions of the Uniformed Services University, the US Army, Navy, Air Force, or the DoD.

## Declaration of competing interest

The authors declare no potential conflicts of interest with respect to the research, authorship, and or publication of this article.

## Laboratory Test Working Group

Andrea Deyrup, M.D, PhD., Co-Chair

Barbara Knollmann-Ritschel, M.D., Co-Chair

Danielle D'Ambrosio, M.D., Editor

Jeannie Muir, M.D., Editor

Teresa Scordino, M.D.

Matthew Kraswoski, M.D., Ph.D.

Liyun Cao, Ph.D.

Kinjal Shah, M.D.

Jennifer Zepf, D.O.

Samuel Grindstaff, M.D.

Ashley Inman, M.D.

Karen Moser, M.D.

Kristin Olson, M.D.

Lynette Parker, M.D.

Aaron Shmookler, M.D.

Joyce Ou, M.D., Ph.D.

Angelica Putnam, M.D.

Luisa Watts, M.D.

Elham Vali Betts, M.D.

Scott Lovitch, M.D., Ph.D.

Kristen Stashek, M.D.

Melina Flanagan, M.D.

Nirupama Singh, M.D., Ph.D.

Eric Suarez, M.D.

Ellen Dudrey, M.D.

Mary Furlong, M.D.

Marta Margeta, M.D., Ph.D.

Adam Wilberger, M.D.

Joanna Chan, M.D.

Amy Lin, M.D.
